# Copy number variations in Friesian horses and genetic risk factors for insect bite hypersensitivity

**DOI:** 10.1186/s12863-018-0657-0

**Published:** 2018-07-30

**Authors:** Anouk Schurink, Vinicius H. da Silva, Brandon D. Velie, Bert W. Dibbits, Richard P. M. A. Crooijmans, Liesbeth Franҫois, Steven Janssens, Anneleen Stinckens, Sarah Blott, Nadine Buys, Gabriella Lindgren, Bart J. Ducro

**Affiliations:** 1 Animal Breeding and Genomics, Wageningen University & Research, P.O. Box 338, 6700 AH Wageningen, the Netherlands; 2 Department of Animal Breeding and Genetics, Swedish University of Agricultural Sciences, P.O. Box 7023, 75007 Uppsala, Sweden; 3 Department of Animal Ecology, Netherlands Institute of Ecology, NIOO-KNAW, 6708 PB Wageningen, the Netherlands; 4 KU Leuven, Department of Biosystems, Livestock Genetics, P.O. Box 2456, 3001 Heverlee, Belgium; 5 Reproductive Biology, Faculty of Medicine and Health Sciences, The University of Nottingham, Leicestershire, LE12 5RD UK

**Keywords:** Copy number variations, Friesian horse, Genome-wide association study, Insect bite hypersensitivity

## Abstract

**Background:**

Many common and relevant diseases affecting equine welfare have yet to be tested regarding structural variants such as copy number variations (CNVs). CNVs make up a substantial proportion of total genetic variability in populations of many species, resulting in more sequence differences between individuals than SNPs. Associations between CNVs and disease phenotypes have been established in several species, but equine CNV studies have been limited. Aim of this study was to identify CNVs and to perform a genome-wide association (GWA) study in Friesian horses to identify genomic loci associated with insect bite hypersensitivity (IBH), a common seasonal allergic dermatitis observed in many horse breeds worldwide.

**Results:**

Genotypes were obtained using the Axiom® Equine Genotyping Array containing 670,796 SNPs. After quality control of genotypes, 15,041 CNVs and 5350 CNV regions (CNVRs) were identified in 222 Friesian horses. Coverage of the total genome by CNVRs was 11.2% with 49.2% of CNVRs containing genes. 58.0% of CNVRs were novel (i.e. so far only identified in Friesian horses). A SNP- and CNV-based GWA analysis was performed, where about half of the horses were affected by IBH. The SNP-based analysis showed a highly significant association between the MHC region on ECA20 and IBH in Friesian horses. Associations between the MHC region on ECA20 and IBH were also detected based on the CNV-based analysis. However, CNVs associated with IBH in Friesian horses were not often in close proximity to SNPs identified to be associated with IBH.

**Conclusions:**

CNVs were identified in a large sample of the Friesian horse population, thereby contributing to our knowledge on CNVs in horses and facilitating our understanding of the equine genome and its phenotypic expression. A clear association was identified between the MHC region on ECA20 and IBH in Friesian horses based on both SNP- and CNV-based GWA studies. These results imply that MHC contributes to IBH sensitivity in Friesian horses. Although subsequent analyses are needed for verification, nucleotide differences, as well as more complex structural variations like CNVs, seem to contribute to IBH sensitivity. IBH should be considered as a common disease with a complex genomic architecture.

**Electronic supplementary material:**

The online version of this article (10.1186/s12863-018-0657-0) contains supplementary material, which is available to authorized users.

## Background

Genome sequence diversity can be present in various forms ranging from single-nucleotide polymorphisms (SNPs) to structural variants such as copy number variation (CNV). CNVs, a term that refers to a change in the number of copies of a genomic segment, are responsible for more sequence differences between individuals than SNPs and are considered to be a major source of inter-individual genetic variation contributing to differences in phenotypes (e.g. [1, 2]). Like SNPs, CNVs can be used to identify associations with genetic diseases and other important complex traits. CNVs explain variable penetrance of Mendelian and polygenic diseases and are responsible for variation in phenotypic expression of complex traits [e.g. 1, 2]. Considerable advances regarding CNVs have been made during the last decade [2].

Several studies identified copy number variations in horses using different techniques [3–12]. Part of these studies tried to establish associations between CNVs and a specific trait, a disease or even gene expression [3, 5–12]. Most of these studies found either no association or inconclusive associations as the number of horses with phenotypic information or with specific CNVs were limited. For example, a 62 kb duplication on *Equus caballus* (ECA) chromosome 10 seemed to be related to recurrent laryngeal neuropathy [5]. However, this duplication was detected in two unphenotyped parents and only 10 out of 234 cases (representing three breeds), but in none of the 228 controls [5]. The number of horses (between 4 to 70) studied by Doan and colleagues [3], Ghosh and colleagues [6], Wang and colleagues [11], Ghosh and colleagues [7], Park and colleagues [10] and McQueen and colleagues [8] was likely too few to be able to establish associations between CNVs and the investigated complex phenotypes. Pawlina-Tyszko and colleagues [12] identified an association between CNVs and equine sarcoids as these structural variants were overrepresented in sarcoid cells compared to unaltered skin tissue samples from 16 horses. Metzger and colleagues [9] performed a CNV-based genome-wide association (GWA) study in 717 horses from 17 breeds and showed that three CNV regions on ECA1, ECA8 and ECA9 were significantly associated with equine body size. To our knowledge, significant associations between CNVs and a specific trait in horses using a genome-wide approach have only been detected by Metzger and colleagues [9], an achievement likely accomplished because they investigated a large sample (*n* = 717) and their trait of interest (body size) may not have been as complex as most of the diseases investigated to date (reflected by a substantial heritability of the trait). Many common and relevant diseases affecting equine welfare and other traits of importance have not yet been tested based on structural variants.

One such disease is insect bite hypersensitivity (IBH), a seasonal allergic dermatitis observed in many horse breeds worldwide. The hypersensitive reaction to bites of *Culicoides* spp. causes an intense itch that results in self-inflicted trauma. Common clinical symptoms are hair loss, thickened skin, scaling and even open wounds (e.g. [13]). The welfare and commercial value of affected horses is therefore seriously reduced. Moreover, owners from affected horses suffer economic losses mostly due to an attempt to alleviate the itch and treat clinical symptoms.

The aetiology of IBH is multifactorial in origin and involves both environmental and genetic factors. Both heritability estimates (e.g. [14, 15]) and previous GWA studies [16–19] showed that the inheritance of IBH is truly polygenic in nature. Several breed-specific loci were identified, while across-breed associations with IBH were located on ECA7, 9, 11 and 20 [16–18]. Several candidate genes have been examined for an association with IBH in various breeds. However, associations with IBH were not often established or were inconsistent across breeds [20–22]. In contrast, the major histocompatibility complex (MHC, or equine lymphocyte antigens (ELA) in horses) class I and II regions on ECA20 were repeatedly associated with IBH in several horse breeds [21–25] and with allergen-specific immunoglobulin E levels against two moulds [26].

Most GWA studies in horses used SNPs, while the identification of potential associations between specific traits and CNVs has been limited [5–12]. To our best knowledge, a potential association between IBH and CNVs has never been studied and CNVs have not yet been identified in Friesian horses. As CNVs are the largest source of genetic variation identified in the horse genome so far [3, 6], CNV-based GWA studies could facilitate the identification of associations between our trait(s) of interest and additional genetic variation that is otherwise undetectable in SNP-based GWA studies. Moreover, genomic regions associated in CNV-based GWA studies may reveal more complex structures underlying phenotypic variation. Therefore, the aim of this study was to identify CNVs and to perform a CNV-based GWA study to identify genomic loci associated with IBH in Friesian horses. A SNP-based GWA study was performed as well to allow for comparison. Identification of genomic regions and genes associated with IBH will increase our knowledge on the aetiology and will allow for more effective selection aimed at decreasing IBH prevalence.

## Methods

### Phenotypes and horses

Data were gathered through an online inquiry containing questions about the horse, the farm and IBH. Questions concerned among other things clinical symptoms, recurrence of symptoms, application of preventive measures and success of preventive measures that were applied. Where necessary, questions were clarified with figures and pictures of horses depicting clinical symptoms on various parts of the body and of different severity. Development of the inquiry was performed in close collaboration with a veterinary expert on diagnosing IBH from the Veterinary Faculty of Utrecht University. Through the inquiry, the necessary information was obtained to discriminate cases and controls. Cases were defined as Friesian horses showing the typical seasonal appearance of IBH clinical symptoms. In case preventive methods were applied, cases were defined as Friesian horses that will develop symptoms when these methods would cease. Controls were defined as Friesian horses free of clinical symptoms. However, in several controls (11.6%) preventive methods were applied (e.g. a blanket to protect the horse from any kind of biting insects). Owners of these horses indicated that, when the preventive methods would cease, the horse would not develop symptoms.

A strict protocol was followed to select the horses for genotyping to increase the reliability of the phenotype. Controls were carefully selected to ensure exposure to *Culicoides* spp. Controls were therefore located on a farm where at least one case was present (82.6% of the controls). Not all controls could be collected from farms with cases present, and the remaining controls were collected from IBH high-risk regions in the Netherlands (e.g. [27]). Although age at onset of IBH varies greatly [e.g. [14, 16]], average age at onset is considered to be between 2 and 4-years-of age. Controls were therefore required to be at least 4-years-of-age and at least one to two years at risk of developing IBH clinical symptoms. Cases showed symptoms preferably during two or more seasons (90.8% of the cases; Additional file 1) and their status was confirmed by a veterinarian (60.0% of the cases). Moreover, both cases and controls were preferably located for two or more years on their current farm to ensure a constant management (91.4% of the horses).

Data consisted of 280 Friesian horses, of which 142 were cases and 138 were controls. All horses originated from the Netherlands. Age and sex distribution was similar for cases and controls (Additional file 1). Also, cases and controls descended from a similar number of ancestors, where 40 sires had both case(s) and control(s) among their offspring (66% of the data).

### DNA extraction, quality and genotyping

DNA was extracted from hair samples that were collected with written permission of the horse’s owner. DNA was extracted following the standard protocol for hair roots using the NucleoSpin® Tissue kit from Bioke (Macherey-Nagel). Concentration of DNA was measured using the Infinite® M200 and normalized to 10 ng/μl using Tecan EVO®. Lack of degradation of DNA was confirmed by electrophoresis, carried out using a 1-ll sample on 1.5% multi-purpose agarose gels in Tris–borate–EDTA buffer at 120 V.

Genotypes were obtained using the Axiom® Equine Genotyping Array containing 670,796 SNPs. Part of the quality control was performed with Axiom™ Analysis Suite 1.1 using the default settings of the *Best Practices Workflow*. *OTV* (Off Target Variants) *Caller* was used to perform a post-processing analysis to identify miscalled clustering and to re-label horses in this cluster as OTV (for 6083 SNPs). Gender and computed gender based on homozygosity of the X-chromosome matched for all horses. Subsequent quality control was performed using PLINK software v1.07 [28, 29]. SNPs with call-rate < 90% and MAF < 5% were discarded. All horses passed the call-rate threshold of ≥90%.

The pedigree (**A**, according to Colleau [30]) and genomic relationships (**G**, according to VanRaden [31]) among horses was calculated with calc_grm software [32]. Four horses caused large discrepancies between pedigree and genomic relationships; their genotype data was therefore discarded. The multidimensional scaling plot (Additional file 2) showed that the horses belonged to one cluster. After all quality checks were performed, the final genotype dataset to perform a SNP GWA study contained 276 horses – 141 cases and 135 controls – and 307,075 SNPs (45.8% of all SNPs present on the array).

### SNP-based GWA study

The significance level of differences in allele frequencies between cases and controls per SNP was calculated using a χ^2^-test with 1df using the *assoc* and *adjust* commands in PLINK software v1.07 [28, 29]. Genomic Control corrected *P*-values were presented. The conservative Bonferroni corrected significance level was 1.63 × 10^− 7^ ($$ =\raisebox{1ex}{$\alpha $}\!\left/ \!\raisebox{-1ex}{$n$}\right. $$, where *α* was the desired significance level being 0.05 and *n* was the number of SNPs (*n* = 307, 075) that were tested).

### CNV calling

To compute values requested for CNV calling, the files denominated ‘*summary*’, ‘*calls*’ and ‘*confidences*’ that were built during SNP genotyping in Axiom™ Analysis Suite 1.1 were used. These files were applied into PennCNV [33–35] function ‘*generate_affy_geno_cluster.pl*’ to generate canonical clusters values [36] for each SNP on the array. Subsequently, the canonical clusters were used into the ‘*normalize_affy_geno_cluster.pl*’ function, which estimated Log R Ratio (LRR) and population frequencies of the B allele (BAF) values for each of the 276 analysed horses. GC content around each SNP (1 Mb window) was estimated using the BEDTools ‘*nuc*’ function [37] that generated the GCModel file. The GCModel file was needed to adjust LRR values for genomic waves by the ‘*genomic_wave.pl*’ function in PennCNV (for genomic waves bias details, see [33]). To infer BAF values for each SNP, we applied the ‘*compile_pfb.pl*’ function on all 276 analysed horses. The file containing these BAF values was needed to call CNVs with a hidden Markov model algorithm in PennCNV [33–35] with the ‘*detect_cnv.pl*’ function using default parameters. All called CNVs were filtered based on minimum length (≥1 kb, technical reasons related to SNP density), LRR SD (≤0.3) and BAF drift (≤0.02). After quality control, CNV information from the 31 autosomal horse chromosomes and 222 horses (111 cases and 111 controls) remained for the subsequent analysis. Most horses (45 out of 54) were removed based on high BAF drift.

### CNV region identification and association

Individual CNVs of the 222 Friesian horses were merged into CNVRs, i.e. genomic regions in the horse genome covering CNVs that overlapped by at least 1 bp [38] using the *CNVR* building process in CNVRuler [39]. To minimize the possibility of overestimating the size of CNVRs, low-density areas were trimmed: the area covered by < 10% of the total contributing CNVs within a CNVR was removed using the *recurrence* option.

A CNV-based case-control association analysis for IBH – based on all CNVs identified in Friesian horses – was performed with CNVRuler [39]. The performed analysis tested for an association between CNVs at a specific CNVR and the disease status (case or control). Separate *P*-values were generated for gains and losses using logistic regression including one principal component as covariates to adjust for population stratification (second and third principal component were not significant, data not shown). In the end, three association analyses were performed e.g. considering only gains, only losses or both gains and losses. That is, the number of cases with a particular gain (a CNV with more than 2 copies within a specific CNVR) was compared to the number of controls with this gain. The second analysis only considered losses (a CNV with less than 2 copies within a specific CNVR), where again the number of cases with a particular loss was compared to the number of controls with this loss. In the analysis concerning both gains and losses, we compared the number of cases having a CNV (either gain or loss) within a specific CNVR with the number of controls having a CNV within this specific CNVR. We did not relate the number of copies to for instance the severity of IBH, among other things because often many horses had the same number of copies.

We graphically represented all copy number events and respective frequencies in grid of panels implemented in the Bioconductor R package *gtrellis* [40]. Due to space limitation all information shown is based on genomic windows of 1 Mb.

### Gene mapping, enrichment and ontology analysis

The overlap between the CNVRs identified in Friesian horses and genes annotated in the horse genome (*Ensembl* – BioMart: http://www.ensembl.org/biomart/martview/ and [41]; EquCab2) was determined and classified into upstream, downstream, equal, encompassing or inside. The enrichment of overlapping genes was performed based on human orthologues available in BioMart. The use of human orthologues is justified due to the still incomplete annotation of genes on the equine genome [6, 9]. Based on these orthologues, the *ClusterProfiler* Bioconductor R package with the *enrichKEGG* function [42] was used to identify KEGG pathways that were overrepresented in CNVRs.

### CNV validation by qPCR

Validation of low frequent CNVs was performed by quantitative real-time PCR (qPCR) (Additional file 3). Low frequent CNVs were selected, as these CNVs are more likely to be false positives compared to CNVs shared by more horses. We selected 12 individual low frequent CNV calls based on incidence, size and state, to sample CNVs from the whole distribution of size and state. Concerning incidence, unique (private) CNVs and those present in 2 horses (all with exactly the same breakpoints) were selected to represent two classes of low frequent CNVs. Concerning size, for each frequency class, 6 CNVs were selected based on the size distribution. A CNV was selected from the minimum, first quadrant, mean, median, third quadrant and maximum part of the distribution. Concerning state, in each frequency class we ensured the inclusion of at least one CNV belonging to each of the common states (0n, 1n and 3n) (Additional file 3).

To determine the quality of DNA samples to be validated, the amount of dsDNA was measured with Qubit® Fluorometer. Subsequently, for each sample we used 4 different concentrations to determine primer efficiency: 15 ng, 7.5 ng, 3.8 ng and 1.9 ng of DNA. Reactions were assembled in a final volume of 12.5 μl, containing 3.75 μl DNA, 6.25 μl 2X reaction buffer MESA Blue from Invitrogen™, 1.25 μl forward primer (2 μM) and 1.25 μl reverse primer (2 μM). Samples with a CNV in a specific region and diploid (2n) reference samples were tested with the designed primersets (Additional file 3). Measurements were performed with the Applied Biosystems® 7500 real-time PCR system. Cycle thresholds (log_2_ Ct) were corrected for primer efficiency. ΔCt was calculated as Ct from the sample with a specific CNV minus Ct of the diploid (2n) reference sample [43]. The reference sample was given by a random horse with 2n state. As it is challenging to determine the exact state of a CNV with data from SNP-arrays, we considered a CNV validated when its state (loss or gain) was equal.

### Identification of novel CNVRs

We determined the overlap between CNVRs identified in the Friesian horse population and equine CNV(R)s already published in scientific literature [3–9, 11, 12]. It allowed us to identify CNVRs that had not been discovered so far (i.e. = novel CNVRs) and CNVRs that are shared across breeds. CNVRs among studies were considered to overlap when at least 1 bp was in common and were considered the same event (that is, the same CNVR) when the mutual overlap was > 70%.

## Results

### SNP-based GWA study

**Fig. 1:**
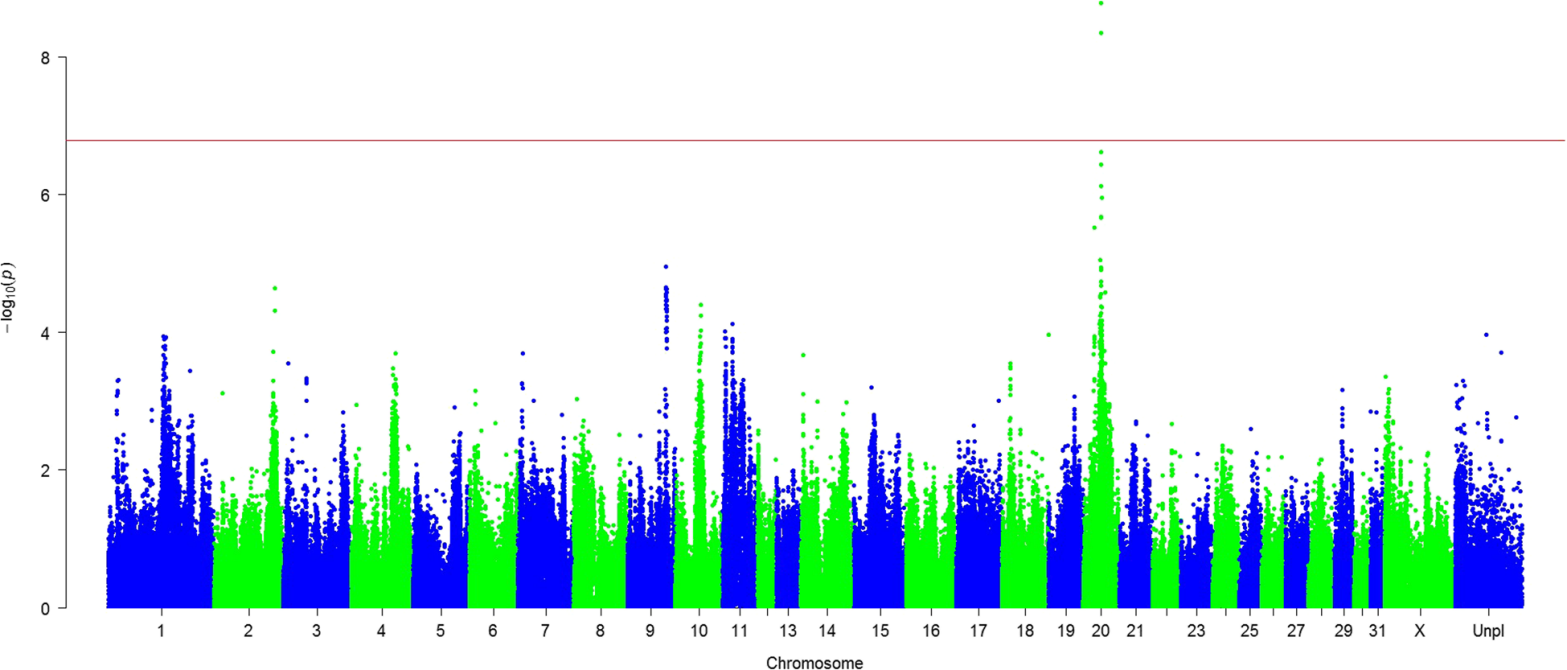
Manhattan plot of insect bite hypersensitivity in Friesian horses based on a SNP-based genome-wide association study. Significance level based on allele frequency differences between cases (*n* = 141) and controls (*n* = 135) using a χ^2^-test (1df). The horizontal red line is the Bonferroni corrected significance level (*P*-value = 1.63 × 10^−7^)

The SNP-based GWA study showed a clear association between a region on ECA20 and IBH in Friesian horses (Fig. 1). Regions with a suggestive association (*P*-value < 0.0001) were identified on ECA2, 9, 10 and 11. In total 2 SNPs passed the Bonferroni corrected significance level (Additional file 4). The SNP most significantly associated with IBH (*P*-value = 7.41 × 10^− 7^) and of good quality was AX-103894624 located on ECA20:31,245,645 (Additional file 5). Estimated odds ratio was 2.62 (allele substitution effect *β* =  *ln* (*OR*) = 0.96). The IBH-associated allele frequency was 0.46 in cases and 0.25 in controls.

### CNV and CNVR detection

**Fig. 2:**
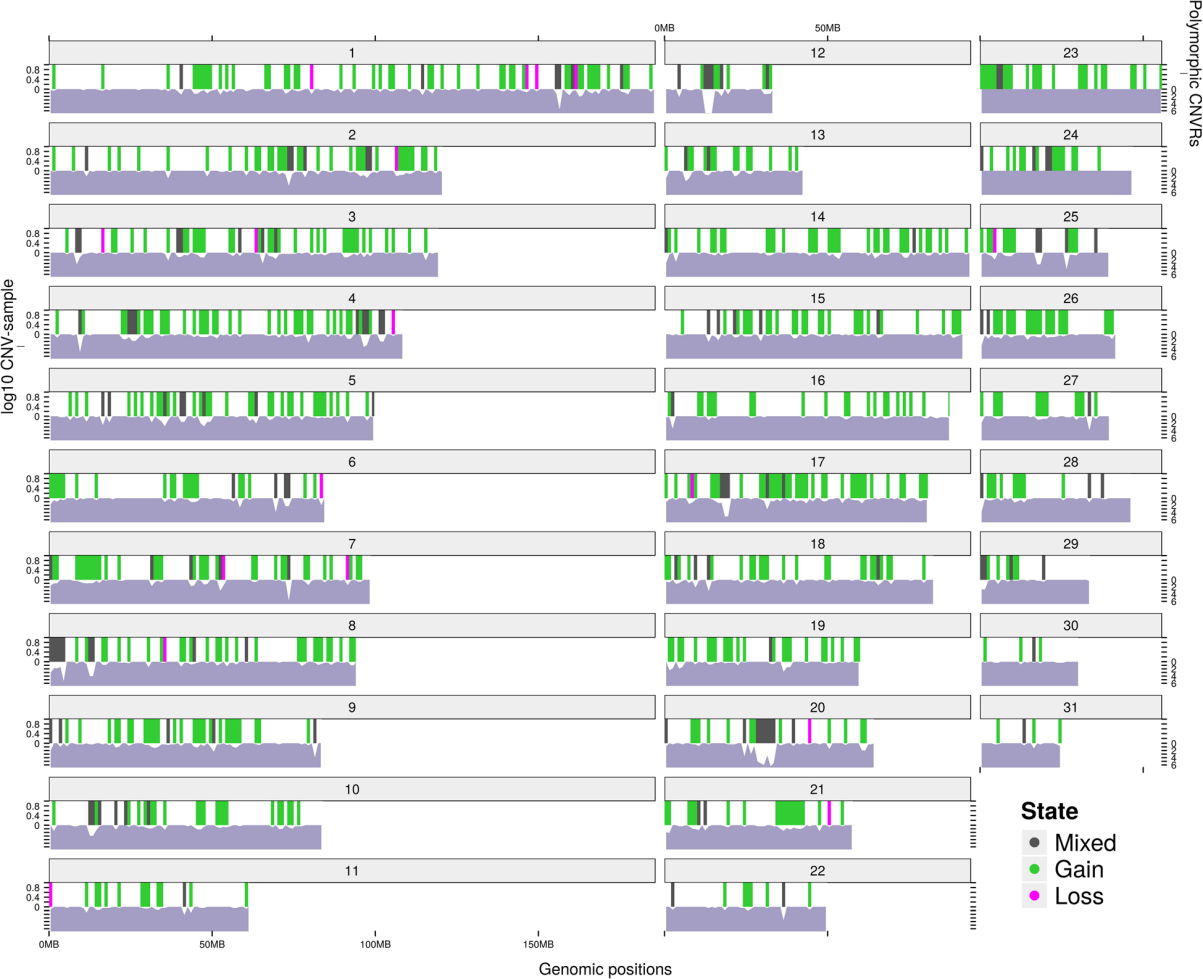
Distribution of polymorphic CNVRs in the Friesian horse genome. Somatic horse chromosomes containing CNV regions with their frequency. The grey bars represent regions harbouring losses and gains concomitantly, the green bars exclusively gains and the magenta bars exclusively losses. The genomic interspersed regions without CNVRs are represented in white. Polymorphic CNVRs: the bars indicate the CNVRs with at least 3 CNVs mapped in 3 different horses (i.e. 1 % of frequency). log10 CNV-sample: the log_10_ of the number of horses containing a CNV event throughout the genome, indicated as a distribution in white

In total, 15,041 CNVs were detected in 222 horses of which 85.5% were gains and 14.5% were losses (Fig. 2, Additional file 6). Number of CNVs per horse ranged from 18 to 262 (mean = 67.8; median = 53.5). Number of SNPs per CNV ranged from 3 to 483 (mean = 15.1; median = 8). Size of the CNVs ranged from 1017 bp to 2.73 Mb (mean = 61.7 kb; median = 29.8 kb). Most CNVs were detected on ECA20, where chromosomal coverage was 11.5% (Additional file 6). CNVs were detected on all autosomes (Additional file 6).

Overlapping CNVs were concatenated into 5350 CNVRs (Additional files 6 and 7). CNVRs encompassing both losses and gains in different horses corresponded to 3.2% (Additional file 6), while only losses corresponded to 2.1% and only gains to 94.7% of all identified CNVRs. Size of the CNVRs ranged from 123 bp to 1.04 Mb (mean = 46.8 kb; median = 29.3 kb). Although the minimum length of a CNV was set to 1 kb, several CNVRs turned out to be < 1 kb due to trimming of low-density areas as described in the Methods section. Chromosomal coverage (percentage of a chromosome covered by CNVRs) was highest for ECA17 (16.2%), followed by ECA26 (16.0%) and ECA12 (15.1%). We did not find a relation between the number of SNPs on a chromosome and chromosomal enrichment for CNVRs (Additional file 6). Coverage of the total genome by CNVRs was 11.2%. In total 1949 CNVRs (36.4%) were shared by at least 2 horses. Mean size of shared CNVRs (79.3 kb) was larger compared to mean size of unique CNVRs (private, 28.2 kb) and 49.2% of CNVRs involved genes (Additional files 6 and 7).

### Association of CNVs with IBH

In total 19 CNVs were associated with IBH (*P*-value < 0.05, Table 1, Additional file 8), but did not reach genome-wide significance. CNVs within a specific CNVR on ECA10 (12,948,489 to 13,075,518 bp) had the lowest *P*-value in the association test (*P*-value = 0.0003), was 127,030 bp in size and included both gains and losses. A CNV within this specific CNVR was observed in 25 cases (15 gains and 10 losses) and 6 controls (all gains) with an odds ratio (OR) of 5.92 (Table 1). CNVs (gains) within a specific CNVR on ECA20 had the lowest *P*-value in the association test based only on gains (*P*-value = 0.001; 30,624,048 to 30,689,273 bp) and was 65,226 bp in size. A CNV (gain) within this specific CNVR was observed in 60 cases and 35 controls with an OR of 2.65 (Table 1). CNVs (losses) within a specific CNVR on ECA20 (30,743,179 to 30,775,429 bp) had the lowest *P*-value in the association test based only on losses (*P*-value = 0.008) and was 32,251 bp in size. A CNV (loss) within this specific CNVR was observed in 18 cases and 5 controls with an OR of 2.45 (Table 1). Individual CNVs within the CNVRs with the lowest *P*-value in the association tests are visualized in Additional file 9. Candidate genes located within CNVRs in which CNVs were associated with IBH are presented in Table 1.

**Table 1:** Results of a CNV-based case-control association analysis of insect bite hypersensitivity in Friesian horses

						CNV frequency										
						Case			Control							
CNVR ID	ECA^a^	Start (bp)	End (bp)	Size (bp)	State	Gain	Loss	Total	Gain	Loss	Total	*P*-value Gain	*P*-value Loss	*P*-value	OR^b^	Gene^c^
CNVR_1144_1	4	23,383,774	23,505,444	121,671	Gain	1	0	1	9	0	9	0.033		0.0332	0.10^0.01–0.84^	
CNVR_1296_1	4	79,687,222	79,957,793	270,572	Gain	11	0	11	26	0	26	0.009		0.0092	0.36^0.17–0.78^	SPAM1
CNVR_2035_1	8	3,638,239	3,783,874	145,636	Mixed	7	2	9	1	1	2	0.046	0.580	0.0367	5.40^1.11–26.3^	IGLV8–61, ENSECAG00000014327
CNVR_2452_1	10	12,948,489	13,075,518	127,030	Mixed	15	10	25	6	0	6	0.022	0.982	0.0003	5.92^2.24–15.7^	AC011513.3, CEACAM1, CEACAM3-CEACAM8, PSG1-PSG9, PSG11
CNVR_2685_1	11	41,743,465	41,832,225	88,761	Mixed	8	5	13	2	2	4	0.055	0.265	0.0245	3.82^1.19–12.3^	
CNVR_2758_1	12	19,366,263	19,527,441	161,179	Gain	14	0	14	2	0	2	0.004		0.0038	9.86^2.09–46.5^	OR4D10, OR4D11, ENSECAG00000008771
CNVR_2979_1	14	64,375,388	64,516,633	141,246	Gain	3	0	3	11	0	11	0.040		0.0395	0.25^0.07–0.94^	
CNVR_4066	20	20,271	503,862	483,592	Mixed	1	0	1	7	1	8	0.064	0.987	0.0450	0.11^0.01–0.95^	
CNVR_4114_1	20	24,230,088	24,296,409	66,322	Mixed	6	0	6	19	0	19	0.010		0.0100	0.28^0.10–0.74^	BTN3A1, BTN3A2, BTN3A3
CNVR_4120_1	20	26,392,472	26,531,248	138,777	Gain	25	0	25	46	0	46	0.003		0.0034	0.41^0.23–0.74^	ENSECAG00000001951, ENSECAG00000019806, ENSECAG00000002019, ENSECAG00000002140, ENSECAG00000002328, ENSECAG00000000865
CNVR_4132_1	20	29,805,544	29,821,078	15,535	Gain	23	0	23	9	0	9	0.007		0.0074	3.11^1.36–7.12^	
CNVR_4140_1	20	30,493,889	30,523,455	29,567	Mixed	34	8	42	56	4	60	0.003	0.248	0.0159	0.52^0.30–0.88^	MICA, MICB
CNVR_4141_1	20	30,624,048	30,689,273	65,226	Gain	60	0	60	35	0	35	0.001		0.0006	2.65^1.52–4.61^	ENSECAG00000017324, ENSECAG00000002838
CNVR_4142_1	20	30,743,179	30,775,429	32,251	Mixed	14	18	32	11	5	16	0.422	0.008	0.0092	2.45^1.25–4.79^	ENSECAG00000019095
CNVR_4155_1	20	32,467,872	32,582,288	114,417	Mixed	3	10	13	7	19	26	0.231	0.086	0.0285	0.44^0.21–0.92^	
CNVR_4271_1	21	10,784,937	10,861,217	76,281	Mixed	8	1	9	3	0	3	0.077	0.987	0.0464	4.20^1.02–17.3^	
CNVR_4537	23	32,331,580	32,716,916	385,337	Gain	1	0	1	9	0	9	0.034		0.0342	0.11^0.01–0.85^	ENSECAG00000026512, ENSECAG00000009426
CNVR_4747^d^	26	250,337	434,632	184,296	Mixed	1	8	9	8	8	16	0.048	0.966	0.0332	0.12^0.01–0.98^	
CNVR_4992_1^d^	28	4428	376,070	371,643	Mixed	3	11	14	12	13	25	0.066	0.620	0.0092	0.21^0.05–0.83^	ENSECAG00000001173, ENSECAG00000003651

Significance level (*P*-value) based on CNV frequency differences between cases (*n* = 111) and controls (*n* = 111) using logistic regression and 1 principal component as covariate, including CNVR identification (CNVR_ID), chromosome, start and end position of CNVR (in base pairs), state (gain, loss or complex), odds ratio (OR) and candidate genes based on *Ensembl* IDs and human orthologues

^a^*Equus caballus* chromosome

^b^Odds ratio of the CNV with 95% confidence interval in superscript

^c^*SPAM1* = sperm adhesion molecule 1 (OMIM:600930), *IGLV8–61* = immunoglobulin lambda variable 8–61 (OMIM:147240), *AC011513.3* = ENSG00000267881, *CEACAM1* = carcinoembryonic antigen-related cell adhesion molecule 1 (OMIM:109770), *CEACAM3* = carcinoembryonic antigen-related cell adhesion molecule 3 (OMIM:609142), *CEACAM4* = carcinoembryonic antigen-related cell adhesion molecule 4 (ENSG00000105352), *CEACAM5* = carcinoembryonic antigen-related cell adhesion molecule 5 (OMIM:114890), *CEACAM6* = carcinoembryonic antigen-related cell adhesion molecule 6 (OMIM:163980), *CEACAM7* = carcinoembryonic antigen-related cell adhesion molecule 7 (ENSG00000007306), *CEACAM8* = carcinoembryonic antigen-related cell adhesion molecule 8 (OMIM:615747), *PSG1* = pregnancy-specific beta-1-glycoprotein 1 (OMIM:176390), *PSG2* = pregnancy-specific beta-1-glycoprotein 2 (OMIM:176391), *PSG3* = pregnancy-specific beta-1-glycoprotein 3 (OMIM:176392), *PSG4* = pregnancy-specific beta-1-glycoprotein 4 (OMIM:176393), *PSG5* = pregnancy-specific beta-1-glycoprotein 5 (OMIM:176394), *PSG6* = pregnancy-specific beta-1-glycoprotein 6 (OMIM:176395), *PSG7* = pregnancy-specific beta-1-glycoprotein 7 (OMIM:176396), *PSG8* = pregnancy-specific beta-1-glycoprotein 8 (OMIM:176397), *PSG9* = pregnancy-specific beta-1-glycoprotein 9 (OMIM:176398), *PSG11* = pregnancy-specific beta-1-glycoprotein 11 (OMIM:176401), *OR4D10* = olfactory receptor family 4 subfamily D member 10, *OR4D11* = olfactory receptor family 4 subfamily D member 11, *BTN3A1* = butyrophilin subfamily 3 member A1 (OMIM:613593), *BTN3A2* = butyrophilin subfamily 3 member A2 (OMIM:613594), *BTN3A3* = butyrophilin subfamily 3 member A3 (OMIM:613595), *MICA* = major histocompatibility complex class I chain-related gene A (OMIM:600169), *MICB* = major histocompatibility complex class I chain-related gene B (OMIM:602436)

^d^Odds ratio of CNV gain is mentioned

The CNV-based GWA study results showed that IBH in Friesian horses is associated with CNVs within specific CNVRs on several autosomes. In total 7 out of 19 CNVRs in which CNVs were associated with IBH were located on ECA20 encompassing the MHC class I, II and III region. These CNVRs identified on ECA20 did correspond to the associated region on the same chromosome identified with the SNP-based GWA study (Table 1 and Additional file 5).

### Biological pathways affected by CNVRs

**Fig. 3:**
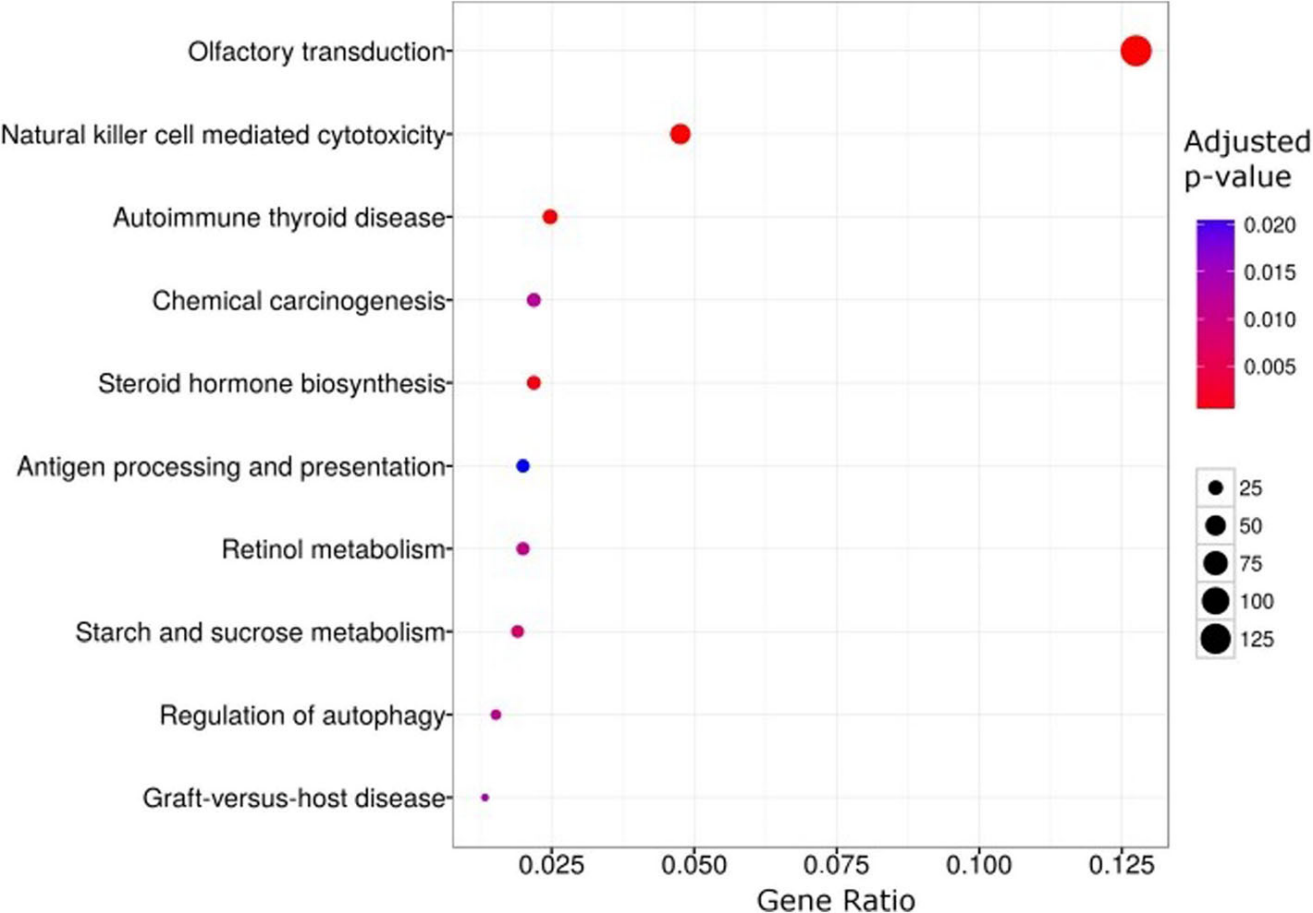
Biological pathways enriched in CNVRs detected in Friesian horses. Biological pathways enriched in CNVRs detected in 222 Friesian horses. To compose the gene enrichment, we used human orthologues (*Ensembl* – BioMart, [41]) of genes overlapping CNVRs. The y-axis indicates the enriched KEGG pathway. The x-axis the ratio between the number of analysed genes and the number of genes in each KEGG pathway presented on the y-axis. The ‘Adjusted *p*-value’ heatmap represents the enrichment *P*-value for each pathway corrected for false discovery rate (FDR, [60]). The circle size represents the number of genes in each pathway

The biological pathways enriched in the identified CNVRs were mainly related to sensory perception, metabolism and immunity (Fig. 3). The pathway with lowest *P*-value was *Olfactory transduction* (*hsa04740*, *P*-value = 1.4 × 10^− 22^) followed by *Natural killer cell mediated cytotoxicity* (*hsa04650*, *P*-value = 1.9 × 10^− 8^) and *Autoimmune thyroid disease* (*hsa05320*, *P*-value = 0.0006). The pathways that we identified were in agreement with previous CNV studies in horses and in other mammals (e.g. [2, 6]), that identified these olfactory receptors, metabolism and immunity related genes as CNV hotspots.

### CNV validation and identification of novel CNVRs

Validation of low frequent CNVs by qPCR indicated that state and copy number of 50% of private CNVs were exactly validated. Also, 66% of the CNVs shared by two horses were validated, where state of the CNV was validated in 50% and state and copy number were validated in 16.7% (Additional file 3).

When looking at the CNVs within 19 CNVRs in Friesian horses significantly associated with IBH, 17 of these regions were also found in one or more horse populations presented in the literature (Additional file 10). Out of these 17 regions, only one was considered a different event as the overlap between the CNVR identified in Friesian horses and the region presented in literature was smaller than 70% (50.2%, CNVR4271; Additional file 10). In total 16 out of 17 CNVRs were considered the same event (that is, the same CNVR) as the region presented in literature. For all these 16 CNVRs, the region identified in Friesian horses completely overlapped with CNV(R)s presented in literature (Additional file 10).

In total 3105 out of 5350 CNVRs were novel (58.0%), that is, so far only identified in the Friesian horse population. 42.0% of the CNVRs were also found in one or more horse populations presented in literature (that is, at least 1 bp overlap).

## Discussion

To date, CNVs have been shown to explain the largest part of genetic variation in genomes of many species including the horse [3, 6]. As such, CNVs are likely to further contribute to our understanding of the equine genome and its expression. Although the efficiency of CNV identification using SNP arrays is lower compared to CNV focussed arrays (e.g. CGH array), the results of the current analyses were comparable to previously published equine CNV studies that predominantly utilized CNV focussed arrays. The chromosomes with the highest CNV distribution (that is, number of CNVs) in this study were ECA1, 12, and 20, the same chromosomes as identified by Dupuis and colleagues [5], Doan and colleagues [3], Metzger and colleagues [9], Ghosh and colleagues [6] and Wang and colleagues [11]. Chromosomes with the greatest coverage by CNVRs were ECA12, 17, and 26, with multiple previous studies also identifying ECA12 [3, 6, 9, 11]. The fraction of the genome covered by CNVRs in our study (11.2%) was higher than previously published by Doan and colleagues [3, 4], Dupuis and colleagues [5], Ghosh and colleagues [6] and Wang and colleagues [11], yet similar to the genome coverage obtained by Metzger and colleagues [9] using PennCNV and 50 k SNP genotyping data. However, CNV and CNVR metrics (e.g. number and size) from equine CNV studies are highly diverse. Ghosh and colleagues [6] composed a CNV dataset for the horse genome based on all published equine CNV data at that time [3–6, 9, 11, 44]. Noticeably, CNV data were included irrespective of the CNV discovery platform used, the analytical methods applied, and the (sometimes limited) number of breeds and individuals. Differences in CNV metrics between the studies also arose from experimental differences [6]. Indeed, the number of CNVs identified in the same horse population differed greatly depending on the CNV detection algorithm that was used, whereas the overlap in detected CNVs was also limited [9]. The composite CNV dataset contains 1476 CNV regions (CNVRs). The majority of these CNVRs were study-specific, while 20% were shared between two or more studies [6].

Recently, data from more contemporary studies were added [7, 8, 10, 12] and indicated that 58.0% of the CNVRs identified in Friesian horses can be considered novel CNVRs. However, Friesian horses are genetically somewhat more inbred and distant to the other investigated breeds as indicated by previous research on genetic diversity in several horse breeds using Short Tandem Repeat loci [45] and SNP data (unpublished data). Therefore, novel CNVRs could be expected and may reflect, in part, variants associated with specific adaptations or with breeding goal traits in Friesian horses. Nonetheless, validation of CNVs is needed in order to conclude if identified CNVs should be considered true structural variants. The state (gain or loss) of low frequent CNVs (private or shared by two horses only) of various lengths was validated for 58.3% of the tested CNVs. Both state and copy number were validated for 33.3% of the tested CNVs. Metzger and colleagues [9] validated state and copy number of 91.7% CNVs through qPCR, but their choice of CNVs to be validated was non-random as only common CNVs associated with body size were selected. Although no attention was paid to incidence, size and state, Pawlina-Tyszko and colleagues [12] randomly selected 12 CNVs using qPCR. Identical validation results were obtained, as both state and copy number were validated for 33.3% of the tested CNVs and state was validated for 58.3%. Validation, characterization and sharing of identified structural variants like CNVs is encouraged as a way to increase our understanding of the equine genome and its expression.

Many traits of interest in horses, as well as common and relevant diseases affecting equine welfare have not been tested in light of structural variants. Only a limited number of studies [3, 5–12] have attempted to detect associations between CNVs and a specific trait, a disease or gene expression in horses. In many studies, no association or inconclusive associations were found, likely due to the number of horses with the phenotype or the specific CNV having been (very) limited. To our best knowledge, we were the first to perform a CNV-based case-control association study for IBH. Most CNVs within specific CNVRs that we found to be associated with IBH were also identified in other horse population(s) [3–9, 11], increasing the likelihood of these CNVs being true structural variants. Several CNVs within or surrounding the major histocompatibility complex region (MHC, or ELA in horses) on ECA20 were associated with IBH in Friesian horses. Some of these CNVs contained MHC class I associated genes (e.g. *BTN3A3*) involved in T cell mediated immunity. Similarly, the SNP-based GWA study and all previous studies on IBH using SNP data to date have identified this region [16, 18, 19, 23]. However, we obtained the lowest *P*-values thus far likely due to an increase in power caused by the large number of inbred horses that were investigated with the high density SNP array combined with a strict protocol to select cases and controls.

Furthermore, serological research established an association between MHC antigens and IBH [24, 25]. MHC class I and II evoke immune responses by recognizing many foreign molecules, whereas MHC class III encodes for complement components and specific cytokines (e.g. TNF-α, [46–48]. The MHC region contains genes that have critical functions in immunity, in both the adaptive and innate immune systems (e.g. [46, 49]). In vertebrates, the MHC region is the most gene-dense region of the genome (e.g. [50, 51]). Genes within this region are extremely polymorphic. In an attempt to explain this unusually high diversity, several hypotheses founded on disease-based and reproductive mechanisms have been formulated (as reviewed by [52, 53]). One such hypothesis is the overdominance (or heterozygote advantage) hypothesis, which states that MHC diversity is maintained by balancing selection for millions of years reflecting “the long-standing battle for supremacy between our immune system and infectious pathogens” (as expressed by [51]). Loss of diversity in these genes seems to reduce the ability to cope with diseases [54] with associations between MHC polymorphisms and fitness having been detected (as reviewed by [52, 53]). Although our knowledge on MHC, its polymorphisms, diversity, and expression continues to increase, there is still much to elucidate. Results of the SNP- and CNV-based GWA studies suggest that the MHC region is associated with IBH. Recently Lanz and colleagues [55] showed that IBH and airway hyperreactivity in horses were associated and discussed the potential of a (partly) common immunogenetic background. The regions identified to be associated with IBH (including MHC) might therefore underlie other equine hypersensitivities as well. Our CNV-based GWA study reflects a first attempt in associating IBH with variants other than SNPs. While validation is still needed to verify the contribution of these CNVs to IBH, it appears that not only nucleotide differences contribute to IBH sensitivity but more complex, structural variations like CNVs also play a role.

Genetic variation is what makes each individual unique and can determine a unique sensitivity to a specific disease. An abundance of SNPs has been identified in many species, and SNPs associated with specific diseases and other phenotypes have been found. Besides the MHC class I, II and III region on ECA20, promising regions likely to hold gene(s) contributing to IBH sensitivity were located on ECA9, 10 and 11 (based on the SNP-based GWA study and [16–19]). However, more in depth research (e.g. sequencing) is needed to be able to identify and validate variations contributing to IBH sensitivity. During the last decade, CNVs proved to be a prevalent form of variation in many species, ultimately responsible for a substantial proportion of the total genetic variability in populations that contributes to a variety of phenotypes. The importance of CNVs has been recognized in modulating gene expression and disease phenotype with associations having been established for autoimmune diseases [56], asthma [57], and obesity [58, 59]. Both CNV and SNP analyses identified an association between the MHC region on ECA20 and IBH (this study and [16–19]). However, CNVs associated with IBH in Friesian horses were not often in close proximity to SNPs previously identified to be associated with IBH. Although this study comprises a first attempt to associate structural variants in the equine genome with IBH, the associated CNVs potentially represent additional regulators of IBH providing further evidence that IBH is a multifactorial polygenic disease with a complex genomic architecture.

## Conclusions

In conclusion, CNVs were identified in a sample of the Friesian horse population and results were comparable to previously published equine CNV research. Characterization and sharing of identified CNVs within various horse breeds, including the Friesian horse breed, contributes to our knowledge on structural variants in horses and enhances our understanding of the equine genome and its phenotypic expression. Results of the association analyses showed that not only do nucleotide differences appear to contribute to IBH sensitivity, but more complex, structural variations like CNVs are also highly likely to be involved. IBH can therefore be considered as a multifactorial polygenic disease with a complex genomic architecture.

## Additional files

Additional file 1: Characteristics of the investigated Friesian horses. Characteristics of the investigated cases concerning preventive methods and the observed, seasonality of clinical symptoms. And characteristics of the investigated cases and controls, where mean age, number of males and females, and pedigree of investigated cases, controls and of the total investigated Friesian horse population are presented. (DOCX 13 kb)

Additional file 2: Multidimensional scaling plot of 276 genotyped Friesian horses. Multidimensional scaling plot of 276 genotyped Friesian horses calculated with *cluster* and *mds-plot* commands in PLINK software v1.07 [28, 29] using autosomal SNPs. (DOCX 31 kb)

Additional file 3: CNVs randomly selected based on incidence and size validated through qPCR. CNVR identification, chromosome (ECA), start and end position (in bp) and size of the CNVs in the investigated Friesians horse sample is presented, including information on whether the CNV concerned a private (present in 1 horse) or shared (present in 2 horses; the exact same breakpoints were observed) CNV. The designed primers, state of the CNV and results of the qPCR are given. (DOCX 14 kb)

Additional file 4: Regional association plot (ECA20) of insect bite hypersensitivity in Friesian horses. Significance level based on allele frequency differences between cases (*n* = 141) and controls (*n* = 135) using a χ^2^-test (1df). The horizontal red line is the Bonferroni corrected significance level (*P*-value = 1.63 × 10^− 7^). (DOCX 27 kb)

Additional file 5: SNPs most significantly associated with insect bite hypersensitivity in Friesian horses. SNPs most significantly associated with insect bite hypersensitivity including chromosome, position (in basepairs), SNP name, *P*-value, odds ratio (OR) and allele frequency in cases and controls. SNPs marked grey passed the Bonferroni corrected significance level (*P*-value = 1.63 × 10^− 7^). (DOCX 14 kb)

Additional file 6:Chromosomal distribution, characteristics and enrichment of detected CNVs and CNVRs. Number of CNVs and CNVRs detected per *Equus caballus* chromosome (ECA), including detection, state, content, mean size (in base pairs), coverage (in base pairs), chromosomal distribution ($=\frac{number\kern0.5em of\kern0.5em CNVs\kern0.5em per\kern0.5em chromosome}{total\kern0.5em number\kern0.5em of\kern0.5em CNVs}\times \kern0.5em 100\%$), chromosomal coverage _(_$\frac{CNVR\kern0.5em coverage\kern0.5em per\kern0.5em chromosome}{length\kern0.5em of\kern0.5em chromosome}\times 100\%$) and SNP coverage in base pairs ($=\frac{length\kern0.5em of\kern0.5em chromosome}{number\kern0.5em of\kern0.5em SNPs\kern0.5em per\kern0.5em chromosome}$). (DOCX 24 kb)

Additional file 7: 5350 CNVRs detected by CNVRuler based on 15,041 CNVs identified by PennCNV in 222 Friesian horses. 5350 CNVRs detected by CNVRuler based on 15,041 CNVs identified in PennCNV [33–35] in 222 Friesian horses. Information that is presented includes CNVR identification (CNVR_ID), *Equus caballus* chromosome (ECA), start position (in bp), end position (in bp), size (in bp), copy number state, number of horses (N) with CNVR and whether the CNVR includes a gene(s) and is present in 1 horse (private) or more (shared). The gene(s) located within the CNVR were identified using human orthologues. Start and end position of the genes annotated in the horse genome are presented, including *Ensembl* IDs and whether the CNVR is upstream, inside, downstream of the gene or encompasses the gene. (XLSX 603 kb)

Additional file 8: Genome-wide CNV and SNP association plots of insect bite hypersensitivity in Friesian horses. Genome-wide CNV and SNP association plots of IBH in 222 Friesian horses. The –log_10_ *P*-value is plotted against the chromosomal location (start position) of CNVs within a specific CNVR and each SNP tested across all chromosomes. The horizontal red line indicates a *P*-value of 0.05 for CNVs within a specific CNVR and the Bonferroni corrected significance level (*P*-value = 1.63 × 10^− 7^) for SNPs. Transparent vertical bars are included to be able to compare the GWA results. A) CNV association plot based on an analysis taking into account both gains and losses. B) CNV association plot based on an analysis taking into account gains only. C) CNV association plot based on an analysis taking into account losses only. D) SNP association plot, for comparison purposes. (DOCX 147 kb)

Additional file 9: Visualization of individual CNVs within the CNVRs with the lowest *P*-value in the association tests. Visualization of individual CNVs within the CNVR on ECA10:12,948,489-13,075,518 (association test including both gains and losses), ECA20:30,624,048-30,689,273 (association test including gains only) and ECA20:30,743,179-30,775,429 (association test including losses only). Each row represents one horse and the X-axis is the position on the chromosome. The black line marks the location of the CNVR. Blue lines represent controls, red lines cases. A dotted line represents a deletion (state = 0n), a striped line represents a CNV with state equals 1n and a solid line represents a duplication (3n). (DOCX 50 kb)

Additional file 10: Overlap between CNVs within specific CNVRs associated with IBH in Friesian horses and CNV(R)s already published in literature. Overlap between CNVs within specific CNVRs associated with IBH (*n* = 19) and CNV(R)s already published in literature. CNVR identification, chromosome (ECA), start and end position (in bp) and size (in bp) of the CNVR is presented. It is indicated how the CNVR in Friesian horses overlapped with the CNV(R)s already published in literature (classification) and how great the overlap of the CNVR in Friesian horses was with literature (in percentage and bp). (DOCX 16 kb)

## Abbreviations

CNV: Copy number variation
CNVR: Copy number variation region
ECA: *Equus caballus*
GWA: Genome-wide association
IBH: Insect bite hypersensitivity
MHC: Major histocompatibility complex
SNP: Single-nucleotide polymorphisms

## References

[1] Beckmann JS, Estivill X, Antonarakis SE (2007). Copy number variants and genetic traits: closer to the resolution of phenotypic to genotypic variability. Nat Rev Genet.

[2] Clop A, Vidal O, Amills M (2012). Copy number variation in the genomes of domestic animals. Anim Genet.

[3] Doan R, Cohen N, Harrington J, Veazey K, Juras R, Cothran G, McCue ME, Skow L, Dindot SV (2012). Identification of copy number variants in horses. Genome Res.

[4] Doan R, Cohen ND, Sawyer J, Ghaffari N, Johnson GD, Johnson CD, Dindot SV (2012). Whole-genome sequencing and genetic variant analysis of a quarter horse mare. BMC Genomics.

[5] Dupuis MC, Zhang Z, Durkin K, Charlier C, Lekeux P, Georges M (2013). Detection of copy number variants in the horse genome and examination of their association with recurrent laryngeal neuropathy. Anim Genet.

[6] Ghosh S, Qu Z, Das PJ, Fang E, Juras R, Cothran EG, McDonell S, Kenney DG, Lear TL, Adelson DL, Chowdhary BP, Raudsepp T (2014). Copy number variation in the horse genome. PLoS Genet.

[7] Ghosh S, Das PJ, McQueen CM, Gerber V, Swiderski CE, Lavoie J-P, Chowdhary BP, Raudsepp T (2016). Analysis of genomic copy number variation in equine recurrent airway obstruction (heaves). Anim Genet.

[8] McQueen CM, Doan R, Dindot SV, Bourquin JR, Zlatev ZZ, Chaffin MK, Blodgett GP, Ivanov I, Cohen ND (2014). Identification of genomic loci associated with *Rhodococcus equi* susceptibility in foals. PLoS One.

[9] Metzger J, Philipp U, Lopes MS, da Camara Machado A, Felicetti M, Silvestrelli M, Distl O (2013). Analysis of copy number variants by three detection algorithms and their association with body size in horses. BMC Genomics.

[10] Park K-D, Kim H, Hwang JY, Lee C-K, Do K-T, Kim H-S, Yang Y-M, Kwon Y-J, Kim J, Kim HJ, Song K-D, Oh J-D, Kim H, Cho B-W, Cho S, Lee H-K (2014). Copy number deletion has little impact on gene expression levels in racehorses. Asian Australas J Anim Sci.

[11] Wang W, Wang S, Hou C, Xing Y, Cao J, Wu K, Liu C, Zhang D, Zhang L, Zhang Y, Zhou H (2014). Genome-wide detection of copy number variations among diverse horse breeds by array CGH. PLoS One.

[12] Pawlina-Tyszko K, Gurgul A, Szmatoła T, Ropka-Molik K, Semik-Gurgul E, Klukowska-Rötzler J, Koch C, Mählmann K, Bugno-Poniewierska M (2017). Genomic landscape of copy number variation and copy neutral loss of heterozygosity events in equine sarcoids reveals increased instability of the sarcoid genome. Biochimie.

[13] van den Boom R, Ducro B, Sloet van Oldruitenborgh-Oosterbaan MM (2008). Identification of factors associated with the development of insect bite hypersensitivity in horses in the Netherlands. Tijdschr Diergeneeskd.

[14] Eriksson S, Grandinson K, Fikse WF, Lindberg L, Mikko S, Broström H, Frey R, Sundquist M, Lindgren G (2008). Genetic analysis of insect bite hypersensitivity (summer eczema) in Icelandic horses. Animal.

[15] Schurink A, Ducro BJ, Heuven HCM, van Arendonk JAM (2011). Genetic parameters of insect bite hypersensitivity in Dutch Friesian broodmares. J Anim Sci.

[16] Schurink A, Wolc A, Ducro BJ, Frankena K, Garrick DJ, Dekkers JC, van Arendonk JAM (2012). Genome-wide association study of insect bite hypersensitivity in two horse populations in the Netherlands. Genet Sel Evol.

[17] Schurink A, Ducro BJ, Bastiaansen JWM, Frankena K, van Arendonk JAM (2013). Genome-wide association study of insect bite hypersensitivity in Dutch Shetland pony mares. Anim Genet.

[18] Shrestha M, Eriksson S, Schurink A, Andersson LS, Sundquist M, Frey R, Broström H, Bergström T, Ducro B, Lindgren G (2015). Genome-wide association study of insect bite hypersensitivity in Swedish-born Icelandic horses. J Hered.

[19] Velie BD, Shrestha M, François L, Schurink A, Tesfayonas YG, Stinckens A, Blott S, Ducro BJ, Mikko S, Thomas R, Swinburne JE, Sundqvist M, Eriksson S, Buys N, Lindgren G (2016). Using an inbred horse breed in a high density genome-wide scan for genetic risk factors of insect bite hypersensitivity (IBH). PLoS One.

[20] Andersson LS, Högström C, Mikko S, Eriksson S, Grandinson K, Broström H, Frey R, Sundquist M, Lindgren G (2009). Polymorphisms in *SPINK5* do not associate with insect bite hypersensitivity in Icelandic horses born in Sweden. Anim Genet.

[21] Klumplerova M, Vychodilova L, Bobrova O, Cvanova M, Futas J, Janova E, Vyskocil M, Vrtkova I, Putnova L, Dusek L, Marti E, Horin P (2013). Major histocompatibility complex and other allergy-related candidate genes associated with insect bite hypersensitivity in Icelandic horses. Mol Biol Rep.

[22] Vychodilova L, Matiasovic J, Bobrova O, Futas J, Klumplerova M, Stejskalova K, Cvanova M, Janova E, Osickova J, Vyskocil M, Sedlinska M, Dusek L, Marti E, Horin P (2013). Immunogenomic analysis of insect bite hypersensitivity in a model horse population. Vet Immunol Immunopathol.

[23] Andersson LS, Swinbune JE, Meadows JRS, Broström H, Eriksson S, Fikse WF, Frey R, Sundquist M, Tseng CT, Mikko S, Lindgren G (2012). The same ELA class II risk factors confer equine insect bite hypersensitivity in two distinct populations. Immunogenetics.

[24] Halldórsdóttir S, Lazary S, Gunnarsson E, Larsen HJ (1991). Distribution of leucocyte antigens in Icelandic horses affected with summer eczema compared to non-affected horses. Equine Vet J.

[25] Marti E, Gerber H, Lazary S (1992). On the genetic basis of equine allergic diseases: II. Insect bite dermal hypersensitivity Equine Vet J.

[26] Eder C, Curik I, Brem G, Crameri R, Bodo I, Habe F, Lazary S, Sölkner J, Marti E (2001). Influence of environmental and genetic factors on allergen-specific immunoglobulin-E levels in sera from Lipizzan horses. Equine Vet J.

[27] van Grevenhof EM, Ducro B, Heuven HCM, Bijma P (2007). Identification of environmental factors affecting the prevalence of insect bite hypersensitivity in Shetland ponies and Friesian horses in the Netherlands. Equine Vet J.

[28] Purcell S. 2007. PLINK, http://zzz.bwh.harvard.edu/plink/.

[29] Purcell S, Neale B, Todd-Brown K, Thomas L, Ferreira MAR, Bender D, Maller J, Sklar P, de Bakker PIW, Daly MJ, Sham PC (2007). PLINK: a toolset for whole-genome association and population-based linkage analysis. Am J Hum Genet.

[30] Colleau JJ (2002). An indirect approach to the extensive calculation of relationship coefficients. Genet Sel Evol.

[31] VanRaden PM (2008). Efficient methods to compute genomic predictions. J Dairy Sci.

[32] Calus MPL (2013). calc_grm – a programme to compute pedigree, genomic, and combined relationship matrices.

[33] Diskin SJ, Li M, Hou C, Yang S, Glessner J, Hakonarson H, Bucan M, Maris JM, Wang K (2008). Adjustment of genomic waves in signal intensities from whole-genome SNP genotyping platforms. Nucleic Acids Res.

[34] Wang K, Li M, Hadley D, Liu R, Glessner J, Grant S, Hakonarson H, Bucan M (2007). PennCNV: an integrated hidden Markov model designed for high-resolution copy number variation detection in whole-genome SNP genotyping data. Genome Res.

[35] Wang K, Chen Z, Tadesse MG, Glessner J, Grant SFA, Hakonarson H, Bucan M, Li M (2008). Modelling genetic inheritance of copy number variations. Nucleic Acids Res.

[36] Peiffer DA, Le JM, Steemers FJ, Chang W, Jenniges T, Garcia F, Haden K, Li J, Shaw CA, Belmont J, Cheung SW, Shen RM, Barker DL, Gunderson KL (2006). High-resolution genomic profiling of chromosomal aberrations using Infinium whole-genome genotyping. Genome Res.

[37] Quinlan AR, Hall IM (2010). BEDTools: a flexible suite of utilities for comparing genomic features. Bioinformatics.

[38] Redon R, Ishikawa S, Fitch KR, Feuk L, Perry GH, Andrews TD, Fiegler H, Shapero MH, Carson AR, Chen W, Cho EK, Dallaire S, Freeman JL, González JR, Gratacòs M, Huang J, Kalaitzopoulos D, Komura D, MacDonald JR, Marshall CR, Mei R, Montgomery L, Nishimura K, Okamura K, Shen F, Somerville MJ, Tchinda J, Valsesia A, Woodwark C, Yang F, Zhang J, Zerjal T, Zhang J, Armengol L, Conrad DF, Estivill X, Tyler-Smith C, Carter NP, Aburatani H, Lee C, Jones KW, Scherer SW, Hurles ME (2006). Global variation in copy number in the human genome. Nature.

[39] Kim J-H, Hu H-J, Yim S-H, Bae JS, Kim S-Y, Chung Y-J (2012). CNVRuler: a copy number variation-based case-control association analysis tool. Bioinformatics.

[40] Gu Z, Eils R, Schlesner M (2016). Gtrellis: an R/Bioconductor package for making genome-level trellis graphics. BMC bioinformatics.

[41] Kasprzyk A. BioMart: driving a paradigm change in biological data management. Database. 2011; 10.1093/database/bar049. 10.1093/database/bar049 PMC3215098 22083790

[42] Yu G, Wang LG, Han Y, He QY (2012). clusterProfiler: an R package for comparing biological themes among gene clusters. OMICS.

[43] D’haene B, Vandesompele J, Hellemans J (2010). Accurate and objective copy number profiling using real-time quantitative PCR. Methods.

[44] Jun J, Cho YS, Hu H, Kim H-M, Jho S, Gadhvi P, Park KM, Lim J, Paek WK, Han K, Manica A, Edwards JS, Bhak J (2014). Whole genome sequence and analysis of the Marwari horse breed and its genetic origin. BMC Genomics.

[45] van de Goor LHP, van Haeringen WA, Lenstra JA (2011). Population studies of 17 equine STR for forensic and phylogenetic analysis. Anim Genet.

[46] Deakin JE, Papenfuss AT, Belov K, Cross JGR, Coggill P, Palmer S, Sims S, Speed TP, Beck S, Graves JAM (2006). Evolution and comparative analysis of the MHC class III inflammatory region. BMC Genomics.

[47] Gustafson AL, Tallmadge RL, Ramlachan N, Miller D, Bird H, Antczak DF, Raudsepp T, Chowdhary BP, Skow LC (2003). An ordered BAC contig map of the equine major histocompatibility complex. Gytogenet Genome Res.

[48] Neefjes J, Jongsma MLM, Paul P, Bakke O (2011). Towards a systems understanding of MHC class I and MHC class II antigen presentation. Nat Rev Immunol.

[49] Trowsdale J (2001). Genetic and functional relationships between MHC and NK receptor genes. Immunity.

[50] Janova E, Matiasovic J, Vahala J, Vodicka R, Van Dyk E, Horin P (2009). Polymorphism and selection in the major histocompatibility complex *DRA* and *DQA* genes in the family Equidae. Immunogenetics.

[51] The MHC sequencing consortium (1999). Complete sequence and gene map of a human major histocompatibility complex. Nature.

[52] Bernatchez L, Landry C (2003). MHC studies in nonmodel vertebrates: what have we learned about natural selection in 15 years?. J Evol Biol.

[53] Jeffery KJ, Bangham CR (2000). Do infectious diseases drive MHC diversity?. Microbes Infect.

[54] Frankham R (2003). Genetics and conservation biology. C.R. Biol.

[55] Lanz S, Brunner A, Graubner C, El M, Gerber V (2017). Insect bite hypersensitivity in horses is associated with airway hyperreactivity. J Vet Intern Med.

[56] Fanciulli M, Norsworthy PJ, Petretto E, Dong R, Harper L, Kamesh L, Heward JM, Gough SC, de Smith A, Blakemore AI, Froguel P, Owen CJ, Pearce SH, Teixeira L, Guillevin L, Graham DS, Pusey CD, Cook HT, Vyse TJ, Aitman TJ (2007). FCGR3B copy number variation is associated with susceptibility to systemic, but not organ-specific, autoimmunity. Nat Genet.

[57] Brasch-Andersen C, Christiansen L, Tan Q, Haagerup A, Vestbo J, Kruse TA (2004). Possible gene dosage effect of glutathione-S-transferases on atopic asthma: using real-time PCR for quantification of GSTM1 and GSTT1 gene copy numbers. Hum Mutat.

[58] Jacquemont S, Reymond A, Zufferey F, Harewood L, Walters RG, Kutalik Z, Martinet D, Shen Y, Valsesia A, Beckmann ND, Thorleifsson G, Belfiore M, Bouquillon S, Campion D, De Leeuw N, De Vries BBA, Esko T, Fernandez BA, Fernandez-Aranda F, Fernandez-Real JM, Gratacos M, Guilmatre A, Hoyer J, Jarvelin MR, Kooy FR, Kurg A, Le Caignec C, Mannik K, Platt OS, Sanlaville D (2011). Mirror extreme BMI phenotypes associated with gene dosage at the chromosome 16p11.2 locus. Nature.

[59] Walters RG, Jacquemont S, Valsesia A, De Smith AJ, Martinet D, Andersson J, Falchi M, Chen F, Andrieux J, Lobbens S, Delobel B, Stutzmann F, El-Sayed Moustafa JS, Chèvre J, Lecoeur C, Vatin V, Bouquillon S, Buxton JL, Boute O, Holder-Espinasse M, Cuisset J, Lemaitre M, Ambresin A, Brioschi A, Gaillard M, Giusti V, Fellmann F, Ferrarini A, Hadjikhani N, Campion D (2010). A new highly penetrant form of obesity due to deletions on chromosome 16p11.2. Nature.

[60] Benjamini Y, Hochberg Y (1995). Controlling the false discovery rate: a practical and powerful approach to multiple testing. J Roy Statist Soc Ser B.

