# Supplementary material for: Copy number variations in Friesian horses and genetic risk factors for insect bite hypersensitivity
Source: BMC Genet. 2018 Jul 30;19:49. doi: 10.1186/s12863-018-0657-0 (PMC6065148; doi:10.1186/s12863-018-0657-0)
Supplement: Supplementary file 1 — Characteristics of the investigated Friesian horses. Characteristics of the investigated cases concerning preventive methods and the observed, seasonality of clinical symptoms. And characteristics of the investigated cases and controls, where mean age, number of males and females, and pedigree of investigated cases, controls and of the total investigated Friesian horse population are presented. (DOCX 13 kb) [file 12863_2018_657_MOESM1_ESM.docx]

### Additional file 1 – Characteristics of the investigated Friesian horses

Characteristics of the investigated cases concerning preventive methods and the observed, seasonality of clinical symptoms.

|  | Percentage |
| --- | --- |
| Preventive measures applied |  |
| Yes | 92.9% |
| No | 7.1% |
| Most commonly applied preventive measures |  |
| Eczema blanket | 81.7% |
| Application oil/cream | 48.1% |
| Anti-insect spray | 31.3% |
| Applied measures successful |  |
| Yes | 77.9% |
| No | 22.1% |
| Number of seasons affected |  |
| 1 season | 9.2% |
| ≥2 seasons | 90.8% |
| Appearance symptoms |  |
| Spring | 71.6% |
| Summer | 26.2% |
| Other | 2.2% |
| Regression of symptoms |  |
| Fall | 48.2% |
| Winter | 50.4% |
| Other | 1.4% |
| Severity of symptoms over years |  |
| Remains equal | 62.0% |
| Differs per year | 17.1% |
| Increases | 14.0% |
| Other | 6.9% |
| Severity of clinical symptoms |  |
| Mild | 62.7% |
| Moderate | 24.5% |
| Severe | 12.8% |
| Most commonly observed symptoms |  |
| Itch | 91.1% |
| Scaling | 57.4% |
| Hair loss | 56.4% |
| Most commonly affected locations |  |
| Mane | 91.2% |
| Tail | 76.5% |
| Head | 38.2% |
| Ears | 32.4% |

Characteristics of the investigated cases and controls. Mean age, number of males and females, and pedigree of investigated cases, controls and of the total investigated Friesian horse population are presented.

| Trait | Case | Control | Total |
| --- | --- | --- | --- |
| Number of horses | 142 | 138 | 280 |
| Age, years |  |  |  |
| Mean (SD) | 11.9 (5.3) | 11.8 (5.0) | 11.8 (5.1) |
| Range | 3 – 26 | 4 – 24 | 3 – 26 |
| Sex |  |  |  |
| Female | 114 | 116 | 230 |
| Male | 28 | 22 | 50 |
| Pedigree |  |  |  |
| Number of sires | 82 | 71 | 113 |
| Number of dams | 132 | 128 | 241 |
