# Supplementary material for: Copy number variations in Friesian horses and genetic risk factors for insect bite hypersensitivity
Source: BMC Genet. 2018 Jul 30;19:49. doi: 10.1186/s12863-018-0657-0 (PMC6065148; doi:10.1186/s12863-018-0657-0)

### Additional file 2 – Multidimensional scaling plot of 276 genotyped Friesian horses

Multidimensional scaling plot of 276 genotyped Friesian horses calculated with *cluster* and *mds-plot* commands in PLINK software v1.07 [28, 29] using autosomal SNPs.


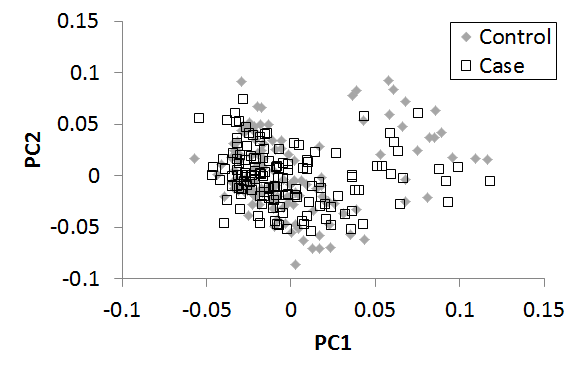

Supplement: Supplementary file 2 — Multidimensional scaling plot of 276 genotyped Friesian horses. Multidimensional scaling plot of 276 genotyped Friesian horses calculated with cluster and mds-plot commands in PLINK software v1.07 [28, 29] using autosomal SNPs. (DOCX 31 kb) [file 12863_2018_657_MOESM2_ESM.docx]
