# Supplementary material for: Copy number variations in Friesian horses and genetic risk factors for insect bite hypersensitivity
Source: BMC Genet. 2018 Jul 30;19:49. doi: 10.1186/s12863-018-0657-0 (PMC6065148; doi:10.1186/s12863-018-0657-0)
Supplement: Supplementary file 3 — CNVs randomly selected based on incidence and size validated through qPCR. CNVR identification, chromosome (ECA), start and end position (in bp) and size of the CNVs in the investigated Friesians horse sample is presented, including information on whether the CNV concerned a private (present in 1 horse) or shared (present in 2 horses; the exact same breakpoints were observed) CNV. The designed primers, state of the CNV and results of the qPCR are given. (DOCX 14 kb) [file 12863_2018_657_MOESM3_ESM.docx]

### Additional file 3 – CNVs randomly selected based on incidence and size validated through qPCR

CNVR identification, chromosome (ECA), start and end position (in bp) and size of the CNVs in the investigated Friesians horse sample is presented, including information on whether the CNV concerned a private (present in 1 horse) or shared (present in 2 horses; the exact same breakpoints were observed) CNV. The designed primers, state of the CNV and results of the qPCR are given.

| CNVR ID | ECA | Start | End | Size | Frequency^1^ | Selection^2^ | State^3^ | FORWARD primer | REVERSE primer | qPCR validation |
| --- | --- | --- | --- | --- | --- | --- | --- | --- | --- | --- |
| CNVR_455 | 2 | 6,604,846 | 6,633,079 | 28,234 | 1 | Private | 3n | CTCATCCTTCCCGATTCAAG | TGACTGAAATTGGTGGGTCA | state and copy number validated |
| CNVR_549 | 2 | 57,911,942 | 58,295,805 | 383,864 | 1 | Private | 3n | ACCACATGGGATCTGGGTTA | AGCTGAGGATGGATGAATGG | not validated, 1n |
| CNVR_1313 | 4 | 85,131,970 | 85,133,058 | 1,089 | 1 | Private | 3n | CAGGCAGAAGAAAAGCAACC | CCATCTCCCATGACTCCATT | not validated, 2n |
| CNVR_1474 | 5 | 42,938,000 | 42,974,425 | 36,426 | 1 | Private | 3n | GAGGAGGAGGATGTGCTGAG | GCGGCCTATCATGTCTCATT | not validated, 1n |
| CNVR_1906 | 7 | 31,308,295 | 31,322,017 | 13,723 | 2 | Shared | 1n | GCTGCATCAATGAGTGAGGA | ACACATACGTCCATGCCTGA | not validated, 3n |
| CNVR_2278 | 9 | 21,363,205 | 21,374,842 | 11,638 | 1 | Private | 1n | CATAGGCCCCTGTTTCTTGA | CTGAGGACATTGCCTTCCAT | state and copy number validated |
| CNVR_2742_1 | 12 | 12,628,967 | 12,638,616 | 9,650 | 2 | Shared | 3n | GTGTATGGGCTCCTGTGGTT | AGTGAGGAGGATGGAGCTGA | not validated, 2n |
| CNVR_2947 | 14 | 50,371,500 | 50,444,641 | 73,142 | 2 | Shared | 3n | CATACGCTTGTCCACCACAC | CCACTCTGGTGAGGGATGTT | state and copy number validated |
| CNVR_3021 | 14 | 76,161,461 | 76,177,420 | 15,960 | 2 | Shared | 3n | ACCAACACTCCCTCCATCAG | AGACCGCAAAGGCTGTAGAA | state (gain) validated, 5n |
| CNVR_3214 | 15 | 65,900,256 | 65,921,640 | 21,385 | 1 | Private | 0n | TGAATTGGACTTGGGGAGAG | TTGCCAGGGTCTGGTTCTAC | state and copy number validated |
| CNVR_3332 | 16 | 46,179,071 | 46,180,799 | 1,729 | 2 | Shared | 3n | GGCGACAGTTTCCAAGGTTA | TGTACAGCAAGGCTCCTGAA | state (gain) validated, 4n |
| CNVR_4758 | 26 | 2,838,859 | 2,851,887 | 13,029 | 2 | Shared | 0n | CCCTCAAGGCACCACTAGAA | GCAGCATCCATACTCCCTGT | state (loss) validated, 1n |

^1^Frequency is the frequency of CNVs concerning the private and shared CNVs.

^2^CNV was present in 1 horse (private) or 2 horses (shared; exact same breakpoints).

^3^0n = deletion of two copies (null), 1n = deletion of one copy (A,B), 3n = single copy duplication (AAA,AAB,ABB,BBB).
