# Supplementary material for: Copy number variations in Friesian horses and genetic risk factors for insect bite hypersensitivity
Source: BMC Genet. 2018 Jul 30;19:49. doi: 10.1186/s12863-018-0657-0 (PMC6065148; doi:10.1186/s12863-018-0657-0)

**Additional file 4 – Regional association plot (ECA20) of insect bite hypersensitivity in Friesian horses**

Significance level based on allele frequency differences between cases (*n* = 141) and controls (*n* = 135) using a χ^2^-test (1df). The horizontal red line is the Bonferroni corrected significance level (*P*-value = 1.63 × 10^-7^).


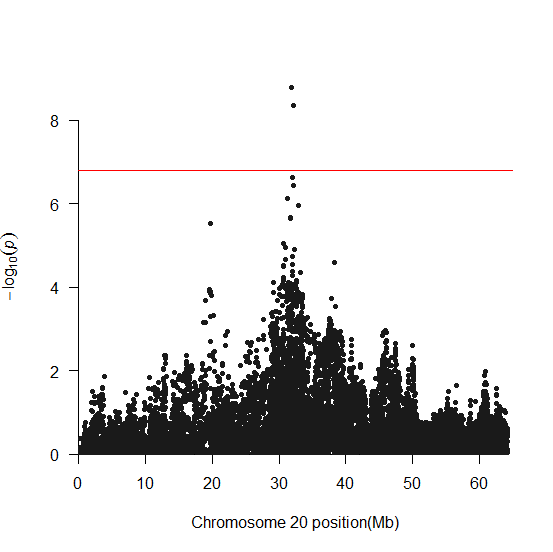

Supplement: Supplementary file 4 — Regional association plot (ECA20) of insect bite hypersensitivity in Friesian horses. Significance level based on allele frequency differences between cases (n = 141) and controls (n = 135) using a χ2-test (1df). The horizontal red line is the Bonferroni corrected significance level (P-value = 1.63 × 10− 7). (DOCX 27 kb) [file 12863_2018_657_MOESM4_ESM.docx]
