# Supplementary material for: Copy number variations in Friesian horses and genetic risk factors for insect bite hypersensitivity
Source: BMC Genet. 2018 Jul 30;19:49. doi: 10.1186/s12863-018-0657-0 (PMC6065148; doi:10.1186/s12863-018-0657-0)
Supplement: Supplementary file 5 — SNPs most significantly associated with insect bite hypersensitivity in Friesian horses. SNPs most significantly associated with insect bite hypersensitivity including chromosome, position (in basepairs), SNP name, P-value, odds ratio (OR) and allele frequency in cases and controls. SNPs marked grey passed the Bonferroni corrected significance level (P-value = 1.63 × 10− 7). (DOCX 14 kb) [file 12863_2018_657_MOESM5_ESM.docx]

## Additional file 5 - SNPs most significantly associated with insect bite hypersensitivity in Friesian horses

SNPs most significantly associated with insect bite hypersensitivity including chromosome, position (in basepairs), SNP name, *P*-value, odds ratio (OR) and allele frequency in cases and controls. SNPs marked grey passed the Bonferroni corrected significance level (*P*-value = 1.63 × 10^-7^).

|  |  |  |  |  | Allele frequency | |
| --- | --- | --- | --- | --- | --- | --- |
| ECA^a^ | Position (bp) | SNP name | *P*-value^b^ | OR^c^ | Cases | Controls |
| 2 | 105,754,941 | AX-103202148^d,e^ | 2.33×10^-05^ | 2.359^1.613-3.449^ | 0.409 | 0.227 |
| 9 | 67,850,426 | AX-104986198 | 2.35×10^-05^ | 0.336^0.206-0.550^ | 0.093 | 0.233 |
| 9 | 68,034,922 | AX-104179213 | 2.36×10^-05^ | 0.354^0.222-0.565^ | 0.106 | 0.252 |
| 9 | 68,079,384 | AX-103384306 | 1.13×10^-05^ | 0.303^0.180-0.512^ | 0.080 | 0.224 |
| 9 | 68,250,373 | AX-104536698 | 2.36×10^-05^ | 0.354^0.222-0.565^ | 0.106 | 0.252 |
| 9 | 68,299,439 | AX-103728993 | 2.24×10^-05^ | 0.356^0.224-0.566^ | 0.110 | 0.258 |
| 20 | 19,706,668 | AX-104183365 | 3.04×10^-06^ | 5.437^2.600-11.37^ | 0.164 | 0.035 |
| 20 | 30,658,219 | AX-103232956^e^ | 8.99×10^-06^ | 2.395^1.657-3.460^ | 0.461 | 0.263 |
| 20 | 31,013,680 | AX-104729989^e^ | 1.14×10^-05^ | 2.298^1.613-3.274^ | 0.519 | 0.319 |
| 20 | 31,042,472 | AX-102963215^e^ | 2.11×10^-05^ | 2.368^1.620-3.460^ | 0.449 | 0.256 |
| 20 | 31,245,645 | AX-103894624 | 7.41×10^-07^ | 2.621^1.819-3.776^ | 0.464 | 0.248 |
| 20 | 31,652,366 | AX-103664602 | 2.19×10^-06^ | 2.420^1.707-3.430^ | 0.518 | 0.307 |
| 20 | 31,655,194 | AX-103592064 | 2.13×10^-06^ | 2.419^1.707-3.427^ | 0.518 | 0.307 |
| 20 | 31,935,993 | AX-104352898^d,e^ | 1.65×10^-09^ | 3.865^2.516-5.938^ | 0.406 | 0.150 |
| 20 | 32,060,910 | AX-103144182 | 1.86×10^-05^ | 0.449^0.317-0.637^ | 0.317 | 0.508 |
| 20 | 32,075,365 | AX-104028000^d,e^ | 2.37×10^-07^ | 2.983^2.000-4.448^ | 0.408 | 0.188 |
| 20 | 32,150,140 | AX-103022378^d,e^ | 3.66×10^-07^ | 2.965^1.979-4.443^ | 0.385 | 0.174 |
| 20 | 32,179,520 | AX-104642494^d,e^ | 4.42×10^-09^ | 4.377^2.681-7.145^ | 0.321 | 0.098 |
| 20 | 32,290,762 | AX-103805671 | 1.24×10^-05^ | 0.427^0.296-0.614^ | 0.286 | 0.484 |
| 20 | 32,941,949 | AX-104318994^e^ | 1.11×10^-06^ | 0.400^0.282-0.569^ | 0.308 | 0.527 |

^a^*Equus caballus* chromosome.

^b^*P*-value corrected for Genomic Control.

^c^Odds ratio of the allele substitution effect of the minor allele within the total population with 95% confidence interval in superscript.

^d^SNP quality is considered low based on its cluster plot.

^e^*P*-value <0.05 for testing Hardy-Weinberg Equilibrium.
