# Supplementary material for: Copy number variations in Friesian horses and genetic risk factors for insect bite hypersensitivity
Source: BMC Genet. 2018 Jul 30;19:49. doi: 10.1186/s12863-018-0657-0 (PMC6065148; doi:10.1186/s12863-018-0657-0)
Supplement: Supplementary file 6 — Chromosomal distribution, characteristics and enrichment of detected CNVs and CNVRs. Number of CNVs and CNVRs detected per Equus caballus chromosome (ECA), including detection, state, content, mean size (in base pairs), coverage (in base pairs), chromosomal distribution ($=\frac{number\kern0.5em of\kern0.5em CNVs\kern0.5em per\kern0.5em chromosome}{total\kern0.5em number\kern0.5em of\kern0.5em CNVs}\times \kern0.5em 100\%$=numberofCNVsperchromosometotalnumberofCNVs×100%), chromosomal coverage ($\frac{CNVR\kern0.5em coverage\kern0.5em per\kern0.5em chromosome}{length\kern0.5em of\kern0.5em chromosome}\times 100\%$CNVRcoverageperchromosomelengthofchromosome×100%) and SNP coverage in base pairs ($=\frac{length\kern0.5em of\kern0.5em chromosome}{number\kern0.5em of\kern0.5em SNPs\kern0.5em per\kern0.5em chromosome}$=lengthofchromosomenumberofSNPsperchromosome). (DOCX 24 kb) [file 12863_2018_657_MOESM6_ESM.docx]

### Additional file 6 – Chromosomal distribution, characteristics and enrichment of detected CNVs and CNVRs

|  | | | |  |  | |  | CNVR characteristics | | | | | | | | | | | | | | |
| --- | --- | --- | --- | --- | --- | --- | --- | --- | --- | --- | --- | --- | --- | --- | --- | --- | --- | --- | --- | --- | --- | --- |
| Chromosome characteristics | | | |  | CNV characteristics | |  |  |  | Detection^1^ | |  | State | | |  | Content^2^ | |  |  |  |  |
| ECA^3^ | Length (bp)^4^ | SNPs | SNP coverage (bp) |  | Number | Distribution (%) |  | Number |  | Shared | Private |  | Gain | Loss | Complex |  | Genic | Intergenic |  | Mean size (bp) | Coverage (bp) | Coverage (%) |
| 1 | 185,838,109 | 61,449 | 3,024 |  | 1,097 | 7.3 |  | 452 |  | 150 | 302 |  | 432 | 14 | 6 |  | 244 | 208 |  | 47,801 | 21,605,974 | 11.6 |
| 2 | 120,857,687 | 52,366 | 2,308 |  | 736 | 4.9 |  | 367 |  | 128 | 239 |  | 351 | 7 | 9 |  | 152 | 215 |  | 35,142 | 12,896,969 | 10.7 |
| 3 | 119,479,920 | 26,730 | 4,470 |  | 626 | 4.2 |  | 284 |  | 110 | 174 |  | 268 | 9 | 7 |  | 126 | 158 |  | 51,833 | 14,720,499 | 12.3 |
| 4 | 108,569,075 | 24,670 | 4,401 |  | 751 | 5.0 |  | 289 |  | 109 | 180 |  | 278 | 5 | 6 |  | 163 | 126 |  | 55,862 | 16,144,118 | 14.9 |
| 5 | 99,680,356 | 23,166 | 4,303 |  | 613 | 4.1 |  | 225 |  | 89 | 136 |  | 210 | 3 | 12 |  | 151 | 74 |  | 53,593 | 12,058,519 | 12.1 |
| 6 | 84,719,076 | 45,517 | 1,861 |  | 515 | 3.4 |  | 236 |  | 60 | 176 |  | 228 | 5 | 3 |  | 112 | 124 |  | 32,662 | 7,708,322 | 9.1 |
| 7 | 98,542,428 | 22,233 | 4,432 |  | 616 | 4.1 |  | 197 |  | 86 | 111 |  | 181 | 9 | 7 |  | 119 | 78 |  | 56,351 | 11,101,068 | 11.3 |
| 8 | 94,057,673 | 20,926 | 4,495 |  | 617 | 4.1 |  | 192 |  | 74 | 118 |  | 179 | 3 | 10 |  | 92 | 100 |  | 53,946 | 10,357,714 | 11.0 |
| 9 | 83,561,422 | 19,272 | 4,336 |  | 472 | 3.1 |  | 214 |  | 83 | 131 |  | 202 | 7 | 5 |  | 89 | 125 |  | 46,469 | 9,944,316 | 11.9 |
| 10 | 83,980,604 | 19,970 | 4,205 |  | 413 | 2.7 |  | 197 |  | 74 | 123 |  | 184 | 7 | 6 |  | 99 | 98 |  | 47,075 | 9,273,687 | 11.0 |
| 11 | 61,308,211 | 14,029 | 4,370 |  | 185 | 1.2 |  | 92 |  | 31 | 61 |  | 87 | 4 | 1 |  | 65 | 27 |  | 48,163 | 4,430,979 | 7.2 |
| 12 | 33,091,231 | 8,787 | 3,766 |  | 1,091 | 7.2 |  | 80 |  | 29 | 51 |  | 65 | 5 | 10 |  | 49 | 31 |  | 62,517 | 5,001,346 | 15.1 |
| 13 | 42,578,167 | 10,388 | 4,099 |  | 199 | 1.3 |  | 56 |  | 28 | 28 |  | 51 | 2 | 3 |  | 40 | 16 |  | 62,621 | 3,506,783 | 8.2 |
| 14 | 93,904,894 | 21,712 | 4,325 |  | 490 | 3.3 |  | 214 |  | 83 | 131 |  | 212 | 0 | 2 |  | 98 | 116 |  | 51,999 | 11127767 | 11.9 |
| 15 | 91,571,448 | 21,091 | 4,342 |  | 371 | 2.5 |  | 200 |  | 72 | 128 |  | 193 | 3 | 4 |  | 94 | 106 |  | 43,054 | 8,610,802 | 9.4 |
| 16 | 87,365,405 | 20,257 | 4,313 |  | 276 | 1.8 |  | 162 |  | 48 | 114 |  | 156 | 3 | 3 |  | 80 | 82 |  | 35,129 | 5,690,941 | 6.5 |
| 17 | 80,757,907 | 18,576 | 4,347 |  | 715 | 4.7 |  | 264 |  | 121 | 143 |  | 257 | 2 | 5 |  | 84 | 180 |  | 49,673 | 13,113,694 | 16.2 |
| 18 | 82,527,541 | 18,767 | 4,397 |  | 549 | 3.6 |  | 233 |  | 80 | 153 |  | 220 | 4 | 9 |  | 120 | 113 |  | 48,520 | 11,305,251 | 13.7 |
| 19 | 59,975,221 | 14,259 | 4,206 |  | 403 | 2.7 |  | 156 |  | 56 | 100 |  | 154 | 1 | 1 |  | 67 | 89 |  | 43,403 | 6,770,897 | 11.3 |
| 20 | 64,166,202 | 37,029 | 1,733 |  | 1,724 | 11.5 |  | 184 |  | 65 | 119 |  | 154 | 6 | 24 |  | 84 | 100 |  | 31,034 | 5,710,226 | 8.9 |
| 21 | 57,723,302 | 13,496 | 4,277 |  | 365 | 2.4 |  | 151 |  | 63 | 88 |  | 146 | 2 | 3 |  | 58 | 93 |  | 43,259 | 6,532,039 | 11.3 |
| 22 | 49,946,797 | 11,707 | 4,266 |  | 160 | 1.1 |  | 84 |  | 18 | 66 |  | 79 | 2 | 3 |  | 41 | 43 |  | 32,746 | 2,750,674 | 5.5 |
| 23 | 55,726,280 | 12,737 | 4,375 |  | 355 | 2.4 |  | 119 |  | 49 | 70 |  | 117 | 0 | 2 |  | 61 | 58 |  | 59,755 | 7,110,823 | 12.8 |
| 24 | 46,749,900 | 10,834 | 4,315 |  | 287 | 1.9 |  | 95 |  | 35 | 60 |  | 84 | 4 | 7 |  | 47 | 48 |  | 52,543 | 4,991,545 | 10.7 |
| 25 | 39,536,964 | 9,320 | 4,242 |  | 270 | 1.8 |  | 66 |  | 26 | 40 |  | 58 | 2 | 6 |  | 48 | 18 |  | 53,314 | 3,518,751 | 8.9 |
| 26 | 41,866,177 | 10,036 | 4,172 |  | 288 | 1.9 |  | 135 |  | 50 | 85 |  | 131 | 2 | 2 |  | 54 | 81 |  | 49,487 | 6,680,781 | 16.0 |
| 27 | 39,960,074 | 9,632 | 4,149 |  | 263 | 1.8 |  | 111 |  | 40 | 71 |  | 106 | 2 | 3 |  | 38 | 73 |  | 48,734 | 5,409,432 | 13.5 |
| 28 | 46,177,339 | 10,675 | 4,326 |  | 241 | 1.6 |  | 119 |  | 40 | 79 |  | 114 | 2 | 3 |  | 63 | 56 |  | 45,319 | 5,392,948 | 11.7 |
| 29 | 33,672,925 | 8,362 | 4,027 |  | 175 | 1.2 |  | 59 |  | 18 | 41 |  | 54 | 0 | 5 |  | 31 | 28 |  | 47,727 | 2815910 | 8.4 |
| 30 | 30,062,385 | 7,507 | 4,005 |  | 93 | 0.6 |  | 68 |  | 18 | 50 |  | 67 | 0 | 1 |  | 38 | 30 |  | 37,748 | 2566878 | 8.5 |
| 31 | 24,984,650 | 6,415 | 3,895 |  | 85 | 0.6 |  | 49 |  | 16 | 33 |  | 48 | 0 | 1 |  | 24 | 25 |  | 35,832 | 1,755,771 | 7.0 |
| Total | 2,242,939,370 | 611,915 |  |  | 15,041 |  |  | 5,350 |  | 1,949 | 3,401 |  | 5,066 | 115 | 169 |  | 2,631 | 2,719 |  |  | 250,605,424 |  |

Number of CNVs and CNVRs detected per *Equus caballus* chromosome (ECA), including detection, state, content, mean size (in base pairs), coverage (in base pairs), chromosomal distribution ($=\frac{\text{number of CNVs per chromosome}}{\text{total number of CNVs}}\times100\%$), chromosomal coverage $=\frac{\text{CNVR coverage per chromosome}}{\text{length of chromosome}}\times100\%$) and SNP coverage in base pairs ($=\frac{\text{length of chromosome}}{\text{number of SNPs per chromosome}}$).

^1^When a CNVR was detected in 2 or more horses, it was considered shared; when a CNVR was detected in 1 horse only, it was considered private.

^2^A CNVR was considered genic when at least a part of the CNVR and gene overlapped.

^3^*Equus caballus* chromosome.

^4^Length of chromosomes in basepairs was retrieved from *Ensembl* (http://www.ensembl.org/Equus_caballus/Info/Index).
