# Supplementary material for: Copy number variations in Friesian horses and genetic risk factors for insect bite hypersensitivity
Source: BMC Genet. 2018 Jul 30;19:49. doi: 10.1186/s12863-018-0657-0 (PMC6065148; doi:10.1186/s12863-018-0657-0)
Supplement: Supplementary file 8 — Genome-wide CNV and SNP association plots of insect bite hypersensitivity in Friesian horses. Genome-wide CNV and SNP association plots of IBH in 222 Friesian horses. The –log10 P-value is plotted against the chromosomal location (start position) of CNVs within a specific CNVR and each SNP tested across all chromosomes. The horizontal red line indicates a P-value of 0.05 for CNVs within a specific CNVR and the Bonferroni corrected significance level (P-value = 1.63 × 10− 7) for SNPs. Transparent vertical bars are included to be able to compare the GWA results. A) CNV association plot based on an analysis taking into account both gains and losses. B) CNV association plot based on an analysis taking into account gains only. C) CNV association plot based on an analysis taking into account losses only. D) SNP association plot, for comparison purposes. (DOCX 147 kb) [file 12863_2018_657_MOESM8_ESM.docx]

**Additional file 8 – Genome-wide CNV and SNP association plots of insect bite hypersensitivity in Friesian horses**

Genome-wide CNV and SNP association plots of IBH in 222 Friesian horses. The –log_10_ *P*-value is plotted against the chromosomal location (start position) of CNVs within a specific CNVR and each SNP tested across all chromosomes. The horizontal red line indicates a *P*-value of 0.05 for CNVs within a specific CNVR and the Bonferroni corrected significance level (*P*-value = 1.63 × 10^-7^) for SNPs. Transparent vertical bars are included to be able to compare the GWA results. A) CNV association plot based on an analysis taking into account both gains and losses. B) CNV association plot based on an analysis taking into account gains only. C) CNV association plot based on an analysis taking into account losses only. D) SNP association plot, for comparison purposes.


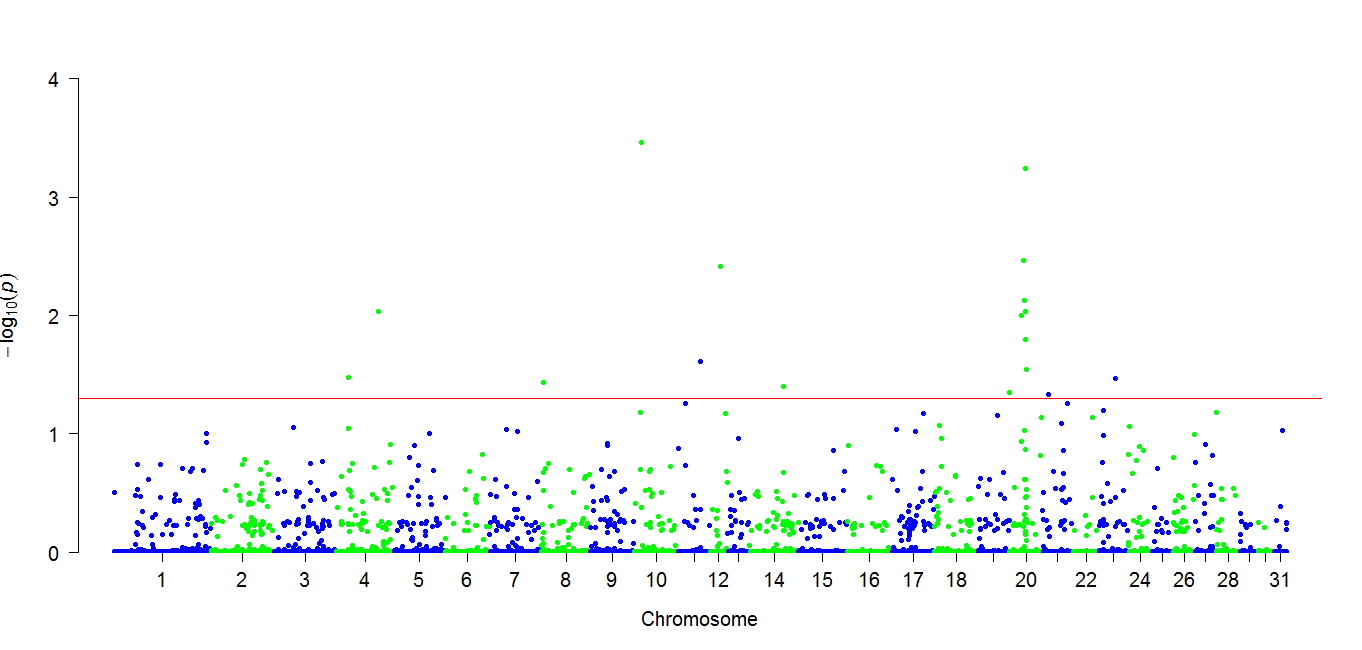


A) A)


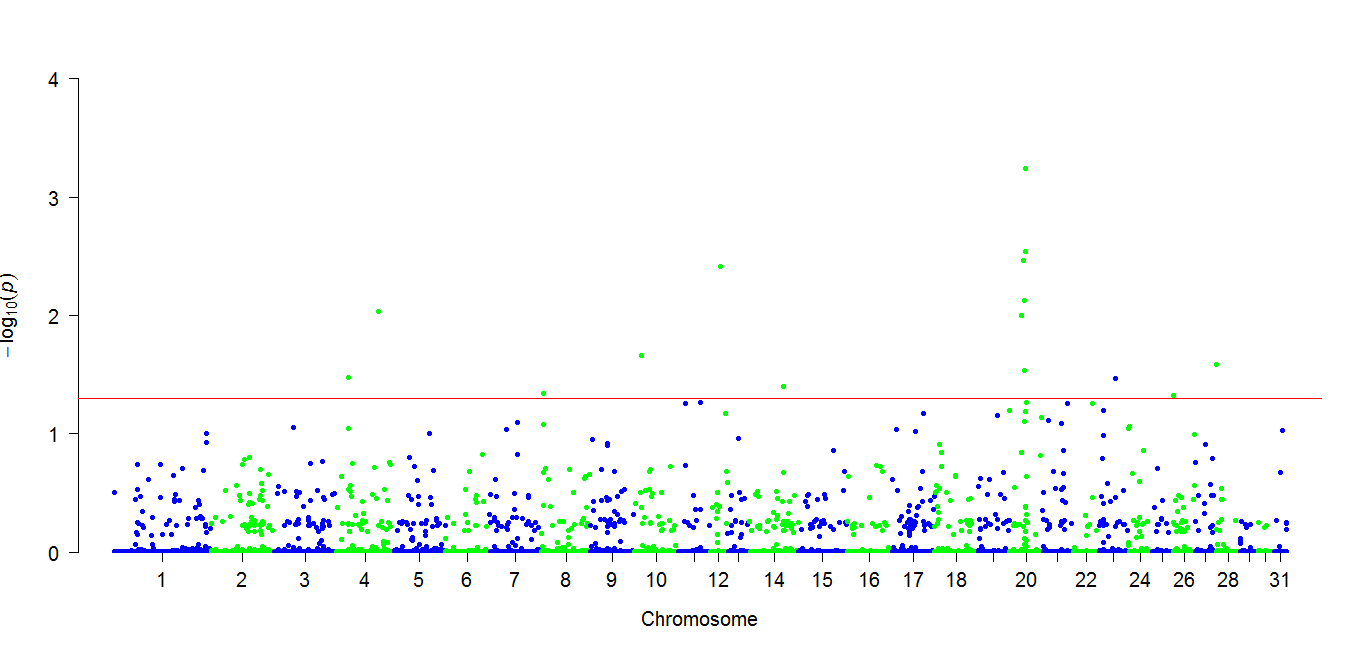

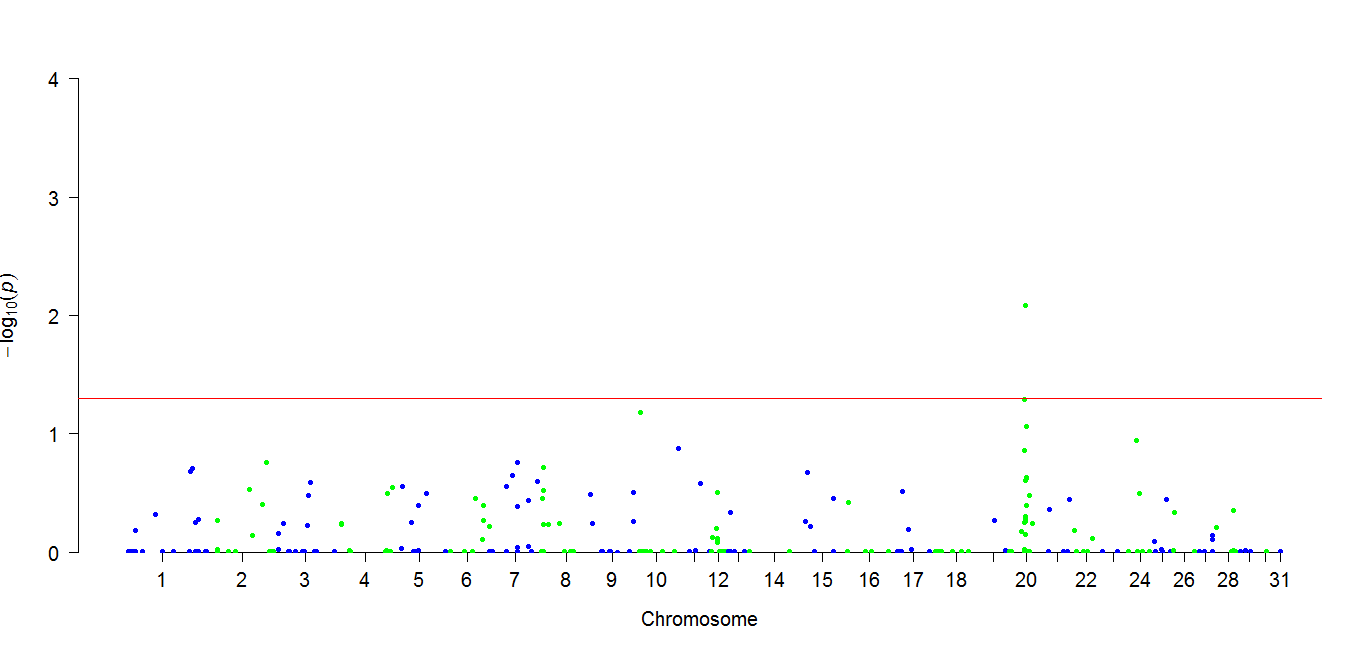

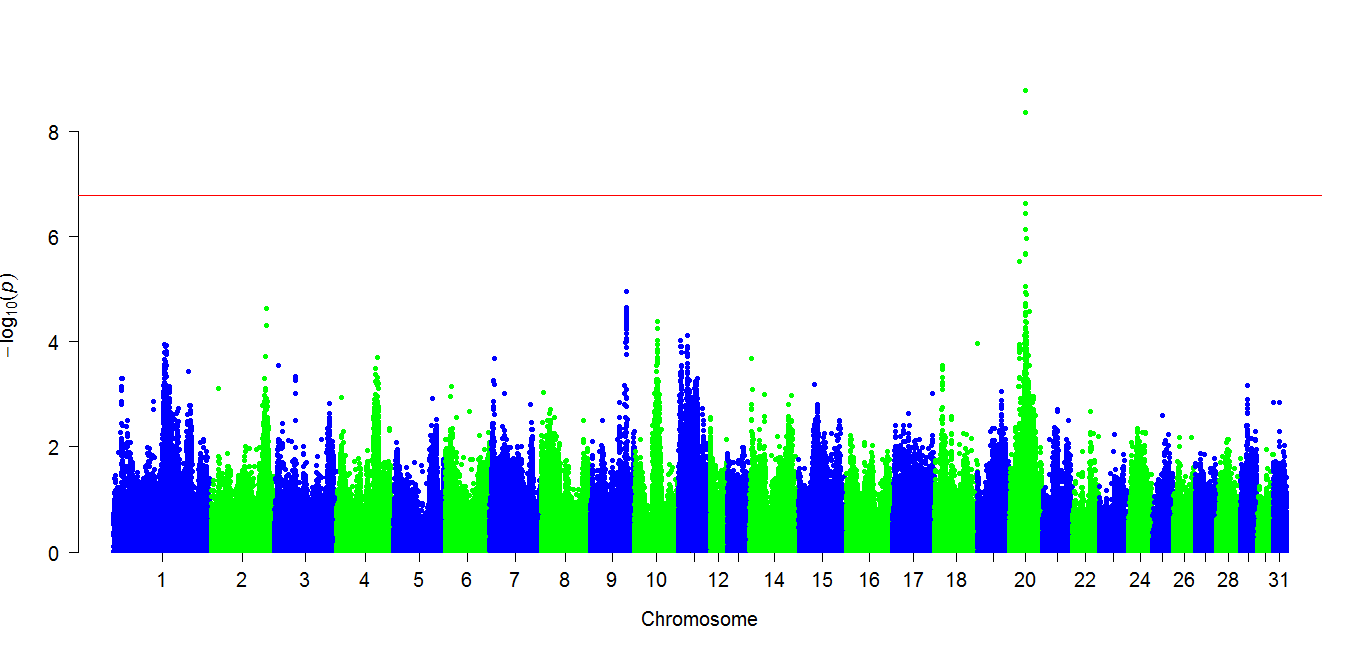


D)A)

C)A)

B)A)

D)A)
