# Supplementary material for: Copy number variations in Friesian horses and genetic risk factors for insect bite hypersensitivity
Source: BMC Genet. 2018 Jul 30;19:49. doi: 10.1186/s12863-018-0657-0 (PMC6065148; doi:10.1186/s12863-018-0657-0)

### Additional file 9 – Visualization of individual CNVs within the CNVRs with the lowest *P*-value in the association tests

Visualization of individual CNVs within the CNVR on ECA10:12,948,489-13,075,518 (association test including both gains and losses), ECA20:30,624,048-30,689,273 (association test including gains only) and ECA20:30,743,179-30,775,429 (association test including losses only). Each row represents one horse and the X-axis is the position on the chromosome. The black line marks the location of the CNVR. Blue lines represent controls, red lines cases. A dotted line represents a deletion (state = 0n), a striped line represents a CNV with state equals 1n and a solid line represents a duplication (3n).


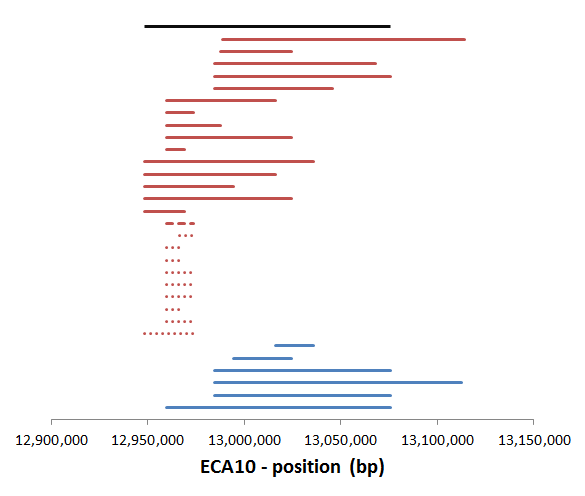


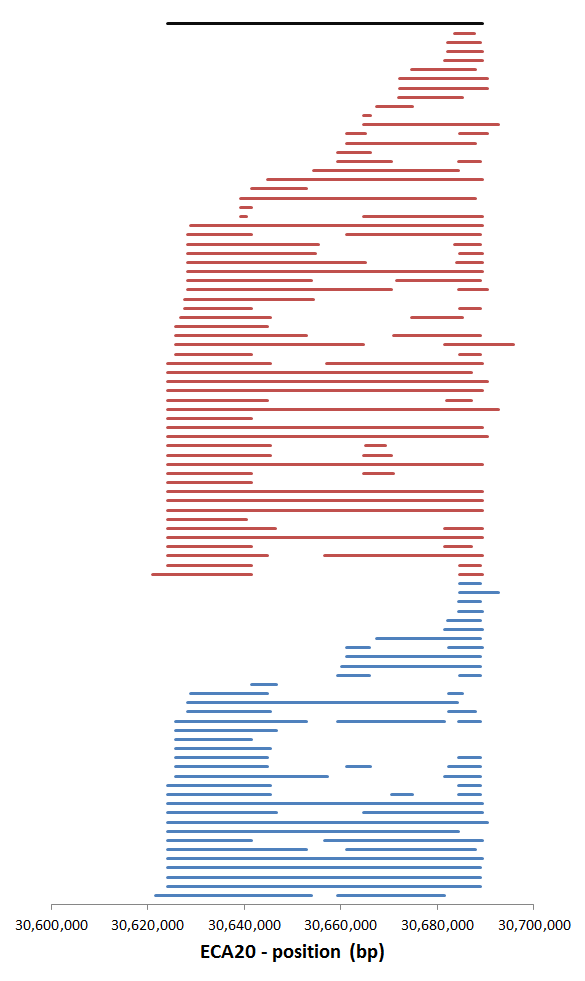


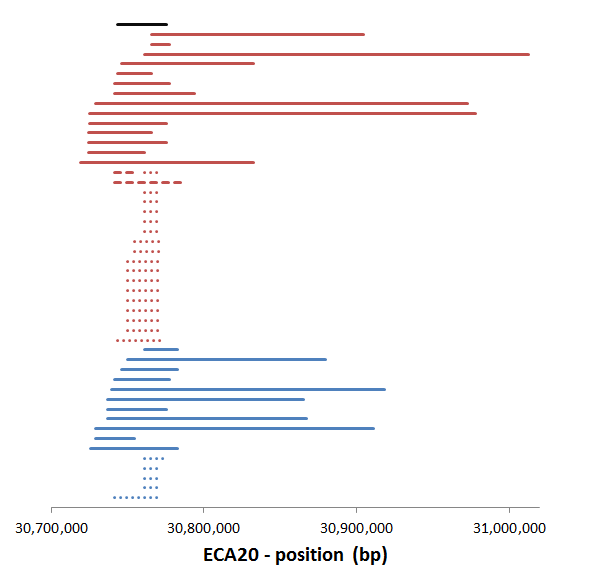

Supplement: Supplementary file 9 — Visualization of individual CNVs within the CNVRs with the lowest P-value in the association tests. Visualization of individual CNVs within the CNVR on ECA10:12,948,489-13,075,518 (association test including both gains and losses), ECA20:30,624,048-30,689,273 (association test including gains only) and ECA20:30,743,179-30,775,429 (association test including losses only). Each row represents one horse and the X-axis is the position on the chromosome. The black line marks the location of the CNVR. Blue lines represent controls, red lines cases. A dotted line represents a deletion (state = 0n), a striped line represents a CNV with state equals 1n and a solid line represents a duplication (3n). (DOCX 50 kb) [file 12863_2018_657_MOESM9_ESM.docx]
