# Supplementary material for: Copy number variations in Friesian horses and genetic risk factors for insect bite hypersensitivity
Source: BMC Genet. 2018 Jul 30;19:49. doi: 10.1186/s12863-018-0657-0 (PMC6065148; doi:10.1186/s12863-018-0657-0)
Supplement: Supplementary file 10 — Overlap between CNVs within specific CNVRs associated with IBH in Friesian horses and CNV(R)s already published in literature. Overlap between CNVs within specific CNVRs associated with IBH (n = 19) and CNV(R)s already published in literature. CNVR identification, chromosome (ECA), start and end position (in bp) and size (in bp) of the CNVR is presented. It is indicated how the CNVR in Friesian horses overlapped with the CNV(R)s already published in literature (classification) and how great the overlap of the CNVR in Friesian horses was with literature (in percentage and bp). (DOCX 16 kb) [file 12863_2018_657_MOESM10_ESM.docx]

### Additional file 10 – Overlap between CNVs within specific CNVRs associated with IBH in Friesian horses and CNV(R)s already published in literature

Overlap between CNVs within specific CNVRs associated with IBH (n = 19) and CNV(R)s already published in literature. CNVR identification, chromosome (ECA), start and end position (in bp) and size (in bp) of the CNVR is presented. It is indicated how the CNVR in Friesian horses overlapped with the CNV(R)s already published in literature (classification) and how great the overlap of the CNVR in Friesian horses was with literature (in percentage and bp).

| CNVR ID | ECA | Start | End | Size | Classification^1^ | Overlap, %^2^ | Overlap, bp |
| --- | --- | --- | --- | --- | --- | --- | --- |
| CNVR_1144_1 | 4 | 23,383,774 | 23,505,444 | 121,671 | Encompass^c,d,f,g^ | 100.0% | 121,671 |
| CNVR_1296_1 | 4 | 79,687,222 | 79,957,793 | 270,572 | Encompass^f^ | 100.0% | 270,572 |
| CNVR_2035_1 | 8 | 3,638,239 | 3,783,874 | 145,636 | Encompass^a,b,c,d,f,g,h^  Downstream^e^ | 100.0%  32.8% | 145,636  47,729 |
| CNVR_2452_1 | 10 | 12,948,489 | 13,075,518 | 127,030 | Encompass^a,b,c,d,g,h^ | 100.0% | 127,030 |
| CNVR_2685_1 | 11 | 41,743,465 | 41,832,225 | 88,761 | Encompass^a,b,c,f,g,h^  Downstream^h^ | 100.0%  0.9% | 88,761  770 |
| CNVR_2758_1 | 12 | 19,366,263 | 19,527,441 | 161,179 | Encompass^a,b,c,d,e,f,h^  Upstream^e^ | 100.0%  0.6% | 161,179  924 |
| CNVR_2979_1 | 14 | 64,375,388 | 64,516,633 | 141,246 | Encompass^a,c,f,g^ | 100.0% | 141,246 |
| CNVR_4066 | 20 | 20,271 | 503,862 | 483,592 | Novel CNVR |  |  |
| CNVR_4114_1 | 20 | 24,230,088 | 24,296,409 | 66,322 | Encompass^a,c,d,e,f,h^  Inside^e,h^  Downstream^e,h^ | 100.0%  2.1%^h^  12.4%^h^  12.7%^h^  53.1%^e^  64.3%^e^  67.0%^h^  94.2%^h^ | 66,322  1,401  8,201  8,439  35,186  42,632  44,419  62,456 |
| CNVR_4120_1 | 20 | 26,392,472 | 26,531,248 | 138,777 | Encompass^a,b,c,d,f,h^  Inside^e,h^  Upstream^e^ | 100.0%  1.0%^h^  1.3%^h^  33.7%^h^  37.1%^e^  38.6%^e^  51.6%^h^  65.0%^e^  87.5%^h^  82.1%  88.0% | 138,777  1,401  1,751  46,700  51,512  53,626  71,635  90,197  121,484  113,946  122,098 |
| CNVR_4132_1 | 20 | 29,805,544 | 29,821,078 | 15,535 | Novel CNVR |  |  |
| CNVR_4140_1 | 20 | 30,493,889 | 30,523,455 | 29,567 | Encompass^a,b,d,e,g,h^  Inside^f^  Upstream^e^ | 100.0%  2.9%  97.1% | 29,567  860  28,709 |
| CNVR_4141_1 | 20 | 30,624,048 | 30,689,273 | 65,226 | Encompass^a,b,d,f,g,h^  Inside^h^  Upstream^h^ | 100.0%  38.6%  29.0% | 65,226  25,160  18,904 |
| CNVR_4142_1 | 20 | 30,743,179 | 30,775,429 | 32,251 | Encompass^a,b,d,f,g,h^ | 100.0% | 32,251 |
| CNVR_4155_1 | 20 | 32,467,872 | 32,582,288 | 114,417 | Encompass^f^  Upstream^a,b,c,d,g,h^ | 100.0%  98.2% | 114,417  112,330 |
| CNVR_4271_1 | 21 | 10,784,937 | 10,861,217 | 76,281 | Downstream^a,b^ | 50.2% | 38,274 |
| CNVR_4537 | 23 | 32,331,580 | 32,716,916 | 385,337 | Encompass^g,h^ | 100.0% | 385,337 |
| CNVR_4747_1 | 26 | 250,337 | 434,632 | 184,296 | Encompass^f^  Inside^h^  Upstream^c,h^ | 100.0%  1.8%  8.6%  6.8%^h^  8.6%^h^  58.4%^c,h^ | 184,296  3,401  15,903  12,503  15,903  107,621 |
| CNVR_4992_1 | 28 | 4,428 | 376,070 | 371,643 | Encompass^b,g,h^  Inside  Upstream^h^  Downstream^h^ | 100.0%  0.5%^h^  0.9%^h^  6.7%^h^  25.9%^e^  74.6%  16.4%  24.5% | 371,643  1,751  3,451  24,898  96,427  277,430  61,010  91,107 |

^1^Encompass = CNVR in Friesian horses is fully captured by the region presented in literature, inside = the region presented in literature is fully captured by the CNVR in Friesian horses, upstream = CNVR in Friesian horses starts upstream of the region presented in literature and ends upstream of the region presented in literature, downstream = CNVR in Friesian horses starts downstream of the region presented in literature and ends downstream of the region presented in literature. Reference to literature: ^a^Doan and colleagues [3]; ^b^Doan and colleagues [4]; ^c^Dupuis and colleagues [5]; ^d^Ghosh and colleagues [6]; ^e^Ghosh and colleagues [7]; ^f^McQueen and colleagues [8]; ^g^Metzger and colleagues [9]; ^h^Wang and colleagues [11].

^2^The percentage of CNVR in Friesian horses that overlaps with the region presented in literature.
